# Laser‐Synthesized Germanium Nanoparticles as Biodegradable Material for Near‐Infrared Photoacoustic Imaging and Cancer Phototherapy

**DOI:** 10.1002/advs.202307060

**Published:** 2024-03-22

**Authors:** Iaroslav B. Belyaev, Ivan V. Zelepukin, Polina A. Kotelnikova, Gleb V. Tikhonowski, Anton A. Popov, Alina Yu. Kapitannikova, Jugal Barman, Alexey N. Kopylov, Daniil N. Bratashov, Ekaterina S. Prikhozhdenko, Andrei V. Kabashin, Sergey M. Deyev, Andrei V. Zvyagin

**Affiliations:** ^1^ Shemyakin‐Ovchinnikov Institute of Bioorganic Chemistry of the Russian Academy of Sciences Moscow 117997 Russia; ^2^ National Research Nuclear University MEPhI (Moscow Engineering Physics Institute) Moscow 115409 Russia; ^3^ Department of Medicinal Chemistry Uppsala University Uppsala 751 23 Sweden; ^4^ Institute of Molecular Theranostics Sechenov University Moscow 119435 Russia; ^5^ Physics Institute Saratov State University Saratov 410012 Russia; ^6^ CNRS LP3 Campus de Luminy – Case 917 Aix Marseille University Marseille Cedex 13288 France; ^7^ MQ Photonics Centre Macquarie University Sydney 2109 Australia

**Keywords:** biodegradable materials, germanium, laser ablation, photoacoustic imaging, photothermal therapy

## Abstract

Biodegradable nanomaterials can significantly improve the safety profile of nanomedicine. Germanium nanoparticles (Ge NPs) with a safe biodegradation pathway are developed as efficient photothermal converters for biomedical applications. Ge NPs synthesized by femtosecond‐laser ablation in liquids rapidly dissolve in physiological‐like environment through the oxidation mechanism. The biodegradation of Ge nanoparticles is preserved in tumor cells in vitro and in normal tissues in mice with a half‐life as short as 3.5 days. Biocompatibility of Ge NPs is confirmed in vivo by hematological, biochemical, and histological analyses. Strong optical absorption of Ge in the near‐infrared spectral range enables photothermal treatment of engrafted tumors in vivo, following intravenous injection of Ge NPs. The photothermal therapy results in a 3.9‐fold reduction of the EMT6/P adenocarcinoma tumor growth with significant prolongation of the mice survival. Excellent mass‐extinction of Ge NPs (7.9 L g^−1^ cm^−1^ at 808 nm) enables photoacoustic imaging of bones and tumors, following intravenous and intratumoral administrations of the nanomaterial. As such, strongly absorbing near‐infrared‐light biodegradable Ge nanomaterial holds promise for advanced theranostics.

## Graphical abstract

Laser‐synthesized germanium (Ge) nanoparticles are presented as material with promising biodegradation behavior. Ge biodegradation proceeds rapidly through oxidation both in vitro and in normal tissues in mice with half‐life of 3.5 days. Strong optical absorption of Ge in the near‐infrared range enables photoacoustic imaging of bones and tumors, as well as photothermal cancer therapy.

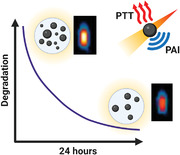

## 1 Introduction

Theranostic nanomedicine has gained momentum due to its versatility, selectivity, and ability to combine imaging and therapeutic functions in one nanoagent. Photoacoustic imaging (PAI) is a hybrid technique that relies on faint ultrasound signal detection upon optical excitation.^[^ ^1^ ^]^ PAI has shown promise for providing morphological and functional information on light‐absorbing tissues and tissues labeled with contrast agents. Photothermal therapy (PTT) is a minimally invasive therapeutic regimen with proven efficiency in humans.^[^ ^2^ ^]^ PTT relies on light‐to‐heat conversion by photosensitizers accumulated in the tumor, which causes cancer cell death locally with minimal damage to surrounding tissues. Recently, PTT using near‐infrared (NIR) light excitation of gold nanoshells demonstrated promising results in clinics for prostate cancer treatment.^[^ ^3^ ^]^

An ideal photosensitizing agent for PAI and PTT must have large light absorption cross‐section, preferably in the optical transparency windows of biological tissues (NIR‐I: 650–950 nm, NIR‐II: 1100–1350 nm),^[^ ^4^ ^]^ along with low toxicity, versatile surface functionalization, and colloidal stability in biological fluids. A set of suitable photosensitizing agents with high NIR‐light absorbance remains exclusive to several nanomaterials, and some of them failed to prove safety after intravenous administration (e.g., gold nanoparticles,^[^ ^5^ ^]^ and carbon nanotubes^[^ ^6^ ^]^). Slow biodegradation and long‐term residence of nanoparticles in the organism tend to cause chronic and unpredictable toxicity unless effective clearance mechanism is in place.^[^ ^7^ ^]^ NIR biophotonics champions, such as gold, iron oxide, and carbon nanoparticles can reside in murine tissues for several months to more than one year.^[^ ^8^, ^9^ ^]^2D nanomaterials exhibit exceptionally high optical mass‐extinction and can be deemed suitable for PAI/PTT applications. However, except for black phosphorus and several MXenes,^[^ ^10^ ^]^ most types of nanosheets are not degradable due to the high crystallinity and low concentration of structural defects.^[^ ^11^ ^]^ Thus, engineering of biocompatible and biodegradable nanoparticles for phototheranostic applications with high optical extinction in NIR range remains challenging.

Germanium material is a strong optical absorber in the NIR‐I and NIR‐II spectral ranges, with a large Bohr radius of 24.3 nm responsible for bandgap photoluminescence in the quantum confinement regime, and effective light‐to‐heat conversion.^[^ ^12^, ^13^, ^14^ ^]^ Furthermore, Germanium belongs to Group IV elements and shares similar chemical properties with silicon, whose biodegradable and optical properties have been utilized for imaging and drug delivery applications.^[^ ^15^ ^]^ The degradation of Ge occurs similar to Si via oxidation and hydrolysis reactions: Ge +  O_2_  → GeO_2_ +  H_2_O  →  H_2_GeO_3_.^[^ ^16^ ^]^ Nevertheless, germanic acid has much higher solubility in water than silicic acid. In addition, unlike silicon, crystalline Ge can be dissolved in acidic environment as in lysosomes of macrophages.^[^ ^17^ ^]^ It is not surprising that a direct comparison of the dissolution rates of crystalline Si and Ge materials shows the faster dissolution of Germanium.^[^ ^18^ ^]^ A range of Germanium‐based nanomaterials was reported to degrade in aqueous solutions, including elemental Ge nanoparticles (Ge NPs),^[^ ^14^, ^19^ ^]^ GeH, and GeP nanosheets.^[^ ^20^, ^21^ ^]^ In most cases, surface erosion accompanied with Germanium oxygenation has been observed. Ge/GeP nanosheets disintegrated into smaller fragments within 9 days of incubation in water,^[^ ^22^ ^]^ while the degradation of GeH nanosheets in physiological‐like buffers was completed within 1 day.^[^ ^20^ ^]^ GeH nanosheets and elemental Ge particles exhibited some degradation in vivo reported by the photoacoustic signal diminution.^[^ ^14^, ^20^ ^]^ Taken together, the biodegradable, optical, and photothermal properties of Germanium materials are deemed very attractive for biomedical applications, provided such critical issues as efficiency and biocompatibility in vivo are addressed.

Here we present a systematic study of rapidly biodegradable spherical Ge nanoparticles and demonstrate their promise as multifunctional theranostic agents. The synthesis of Ge nanoparticles was performed by a method of pulsed laser ablation in liquids, followed by their surface coating with albumin to improve stability and biocompatibility. Rapid hour‐scale dissolution in acidic and neutral pH buffers proceeded via the oxide passivation of the nanoparticle surface. The biodegradability pathway of the nanoformulated Ge showed negligible toxicity in vitro and the absence of chronic toxic effects after intravenous administration in mice. Ge nanoparticles were demonstrated suitable for photoacoustic imaging and photothermal treatment of EMT6/P adenocarcinoma tumors under NIR‐I laser irradiation. We believe that the reported results speak strongly in favor of the application of Germanium nanomaterials for the next‐generation photonic nanomedicine.

## 2 Results

### 2.1 Synthesis and Characterization of Ge Nanoparticles

**Figure 1:**
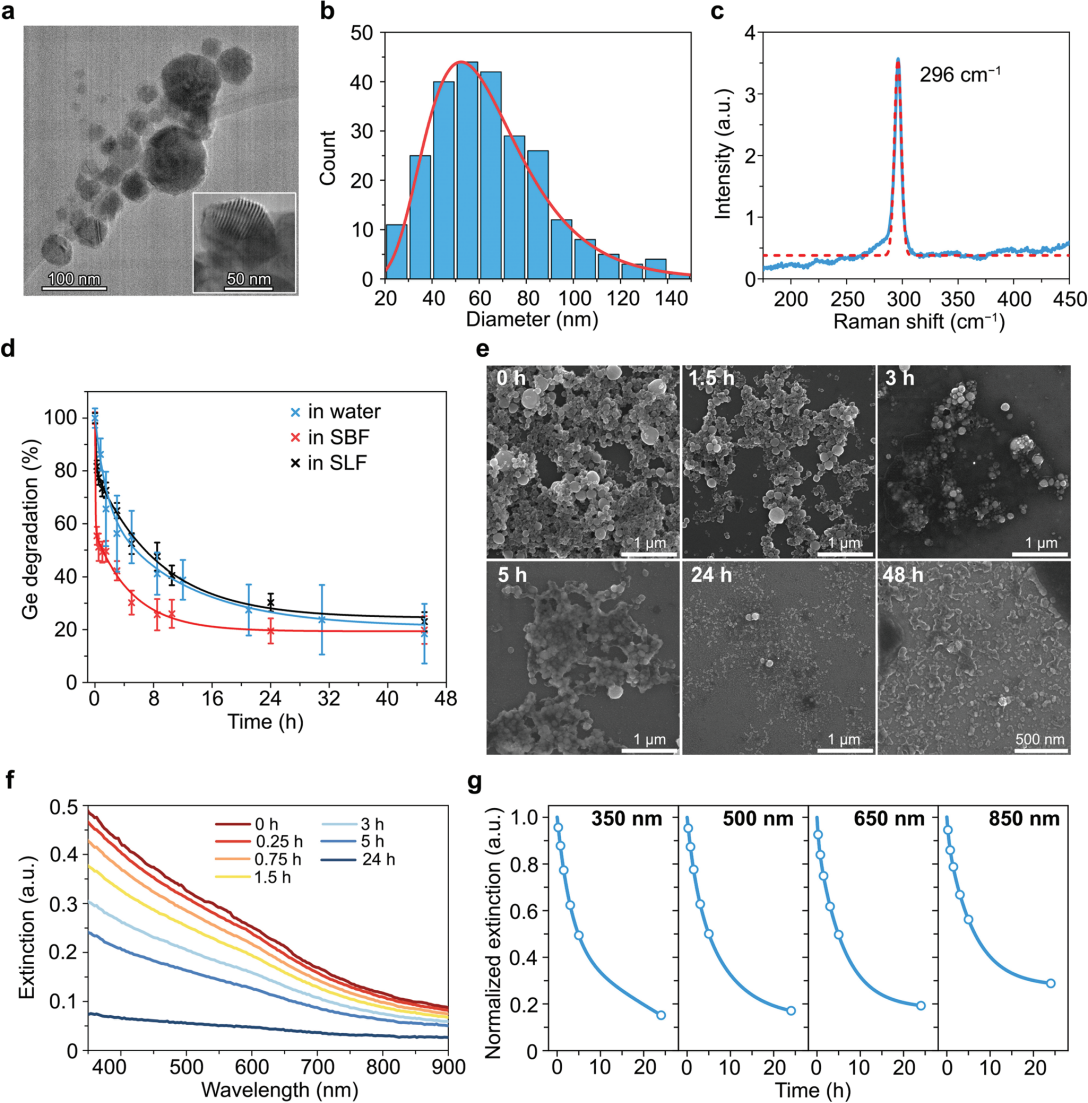
Synthesis and biodegradation of Ge nanoparticles. a) HR‐TEM image of Ge nanoparticles. Inset shows zoomed Moiré fringes on a particle. b) Size distribution of Ge NPs determined from HR‐TEM images. Red line shows log‐normal fitting. n = 250 particles. c) Raman spectrum of Ge nanoparticles. Red line shows Gaussian fitting. d) Ge nanoparticle degradation kinetics in water, simulated body fluid (SBF), and simulated lysosomal fluid (SLF), measured by ICP‐MS. Data are presented as mean ± SD. Solid lines show fitting with biexponential functions. n = 3 samples. e) Representative SEM images of Ge nanoparticles incubated in water for different periods from 0 to 48 h. f) Evolution of optical extinction spectra of Ge NPs during their incubation in water. n = 1 sample. g) Decay kinetics of the optical extinction, measured at 350, 500, 650, and 850‐nm wavelengths. n = 1 sample. Data were normalized to the extinction intensity at 0‐h time point. Solid lines show data fitting with biexponential functions.

Germanium nanoparticles were synthesized by ablation of a germanium wafer in acetone with a high‐energy pulsed femtosecond laser (Figure S1, Supporting Information). The as‐synthesized Ge particles had spherical shape and partially crystalline cores, as indicated by the presence of bright Moiré fringes in high‐resolution transmission electron microscopy (HR‐TEM) images (**Figure** **1**a). The particle size distribution followed a lognormal distribution, with a mean diameter of (67 ± 28) nm (Figure 1b). It is worth noting that the high‐power laser ablation in the femtosecond pulsed regime yielded Ge particle size distribution larger than that produced by the laser ablation operated in the nanosecond (8–18 nm)^[^ ^23^ ^]^ and picosecond (3–43 nm)^[^ ^19^, ^24^ ^]^ pulsed regimes.

The Raman spectrum of Ge NPs (Figure 1c) exhibited an intense peak at 296 cm^−1^ with a full width at half maximum of 7.2 cm^−1^, which was attributed to the transverse mode of phonon oscillations of Ge‐Ge in the crystal lattice. The intensities at the characteristic shifts of GeO_2_ crystalline phase (212, 261, and 440 cm^−1^)^[^ ^25^ ^]^ were comparable to the background level, indicating that as‐synthesized particles in acetone were not significantly oxidized. A shoulder in the range of 260–285 cm^−1^ indicated the presence of a small fraction of amorphous Germanium. Amorphous phase is typical for laser‐ablated nanoparticles.^[^ ^23^, ^26^ ^]^

### 2.2 Ge Nanoparticle Degradation in Aqueous Solutions

The degradation of Ge NPs in aqueous solutions has been reported to proceed through an oxidation mechanism.^[^ ^13^, ^19^ ^]^ We investigated the dissolution of Ge NPs in water and buffers, which simulated relevant physiological environments.^[^ ^27^ ^]^ Ge NPs were incubated in solutions in 100 mg L^−1^ concentration at room temperature and Ge quantities were measured at several time points by means of inductively coupled plasma mass‐spectrometry (ICP‐MS).

Approximately a 45% mass loss of germanium mass was observed after 5 h of the particle incubation in water. Then the degradation rate slowed down, resulting in a 61% and 82% Ge release at 12 and 45 h, respectively. The degradation of Ge NPs was significantly accelerated in simulated body fluid (SBF, 246 mm, pH 7.4), with 49% and 80% of dissolution after 30 min and 45 h, respectively (Figure 1d). In simulated lysosomal fluid (SLF, 322 mm, pH 4.5), the degradation was similar to that of water with 48% of Ge NP loss observed 5 h post incubation, followed by 77% degradation after 45 h. Distilled water was characterized by pH of 5.5 due to the dissolution of CO_2_ from air, tending to further acidification due to germanic acid formation as Ge NPs degraded. Thus, Ge nanoparticles degradation greatly depends on the pH of media, slowing down in acidic environment. Similar behavior was observed for silicon particles, where hydroxyl anions excess accelerated the surface hydration and release of silicic acid.^[^ ^28^ ^]^

To evaluate how complex salt ions composition influences the degradation of Ge particles, we analyzed its dissolution in citrate buffer (100 mm, pH 4.5) and phosphate‐buffered saline (100 mm, pH 7.4), which had simpler compositions and lower salt concentrations. The degradation behavior of Ge NPs in citrate buffer and PBS was identical to the degradation in SLF and SBF buffers, respectively (Figure S2a,b, Supporting Information). This result indicated that ionic strength is unlikely have strong influence on dissolution, while pH remains main driving force of the process.

Then we analyzed how Ge NPs degradation progressed in cell DMEM/F12 medium (pH 7.4) in presence or absence of 10% fetal bovine serum (FBS). Although the as‐procured medium contained glucose, vitamins, and amino acids, the Ge NP degradation rate changed insignificantly in comparison with that of the SBF buffer. On the contrary, presence of serum proteins slowed down the Ge dissolution (Figure S2c, Supporting Information). The 50% Ge degradation level was observed within 2 h for DMEM and within 5 h for DMEM with FBS. Then, at 45‐h timepoint, 82% and 77% of Ge NPs were dissolved in DMEM and DMEM+FBS, respectively. We believe that the formation of a corona from serum proteins stabilized the Ge NP surface and protect particles from degradation.

To investigate the mechanism of Ge NP degradation, we employed scanning electron microscopy (SEM) to observe temporal variation of the nanoparticle morphology in water. The concentration of discrete Ge NPs with a spherical shape steadily decreased over time, while particle residues formed shapeless structures after 24 h of incubation (Figure 1e). Energy‐dispersive spectroscopy coupled with SEM revealed an increase of O/Ge ratio in the Ge NPs over time (Figure S3, Supporting Information). The Ge nanomaterial transformation to amorphous oxidized Germanium was completed by 48 h post incubation. These changes were confirmed by Raman spectroscopy indicating a decrease in the intensity of the peak at 300 cm^−1^ characteristic for Ge crystalline phase, but lacking peaks of crystalline GeO_2_ (Figure S4, Supporting Information). These observations indicated that oxidation and hydrolysis of nanocrystalline Ge led to its amorphization and eventually dissolution. GeO_2_ is known to be moderately soluble in water (10.7 g L^−1^), while pure Ge only dissolves in strong basic or acidic solutions.^[^ ^29^ ^]^

Optical extinction spectra of Ge NPs in solutions were acquired by UV–vis spectroscopy to further investigate the dissolution kinetics. A monotonic decrease in the optical extinction of aqueous solutions of Ge NPs was observed over time (Figure 1f; Figure S5, Supporting Information), which indicated the sample dissolution. Decay kinetics of the extinction was similar in the UV and visible spectral ranges (a 50% drop at 5 h post incubation) and slowed down in the NIR‐I range (Figure 1g). We infer that the larger and more oxidized Ge particles obtained after the degradation rendered the larger optical extinction cross‐section in the NIR‐I region. This effect can be beneficial for optical applications such as PAI and PTT, as the photothermal properties of Ge particles persist or deteriorate slowly, as the dissolution progresses.

### 2.3 Colloidal Stabilization of Ge Nanoparticles

To improve the biocompatibility and colloidal stability of Ge NPs in physiological conditions, we utilized a surface coating strategy by co‐incubation of Ge nanoparticles with bovine serum albumin (BSA). BSA has been frequently used for nanoparticle colloidal stabilization in drug delivery approaches due to protein hydrophilicity, high abundance in mammalian plasma, and high structural similarity of BSA to human serum albumin.^[^ ^30^ ^]^

**Figure 2:**
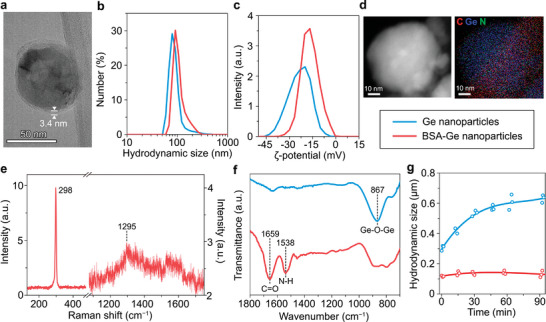
BSA‐coating of Ge nanoparticles. a) Representative HR‐TEM image of BSA‐coated Ge NPs. Scale bar – 50 nm. b) Hydrodynamic particle size distribution of BSA‐Ge NPs in water. *n* = 1 sample. c) ζ‐potential distribution of Ge NPs and BSA‐Ge NPs in 10‐mm NaCl solution. *n* = 1 sample. d) Energy‐dispersive X‐ray mapping analysis of BSA‐Ge nanoparticle. Scale bars – 10 nm. Carbon, nitrogen, and germanium elements are labeled with red, green, and blue colors, respectively. e) Raman spectrum of BSA‐Ge NPs. *n* = 1 sample. f) Fourier‐transform infrared spectra of Ge NPs and BSA‐Ge NPs. *n* = 1 sample. g) Time evolution of the mean hydrodynamic diameters of Ge NPs and BSA‐Ge NPs in PBS buffer. *n* = 3 samples.

After the coating, a low electron density layer was observed on the surface of the Ge particles by HR‐TEM attributed to the presence of organic molecules (**Figure** **2**a). The protein quantity on the surface of NPs was measured to be (73 ± 16) mg of BSA per 1 g of particles, according to the Bradford assay. The mean hydrodynamic diameter of Ge NPs was increased from (91 ± 37) nm to (109 ± 35) nm in water likely due to dissolution of smaller nanoparticles during the coating procedure (Figure 2b). After the coating mass yield of the germanium particles was 63%, as determined by ICP‐MS. BSA‐Ge NPs were characterized by a narrower ζ‐potential distribution of (−17 ± 6) mV (Figure 2c), indicating improved surface homogeneity after the coating. Energy‐dispersive spectroscopy coupled with TEM revealed a large amount of nitrogen and carbon atoms likely from proteins in BSA‐Ge NPs (Figure 2d). Raman spectroscopy confirmed that the particle crystallinity was preserved after the BSA coating, while its protein content was indicated by the emergence of low‐intensity bands in the amide region of 1200–1700 cm^−1^, with a major peak at 1295 cm^−1^ assigned to C─N and N─H groups vibrations (Figure 2e).^[^ ^31^ ^]^ Fourier transformed infrared (FTIR) analysis of BSA‐Ge NPs confirmed the surface immobilization of BSA (Figure 2f), as indicated by the presence of characteristic to proteins amide‐I (C═O stretching at 1659 cm^−1^) and amide‐II (N─H bending and C─N stretching at 1538 cm^−1^) peaks. A Ge‐O‐Ge stretching band at 867 cm^−1^ attributed to oxidized germanium was observed in the BSA‐Ge NP sample and indicated dissolution propensity of the nanoparticles.

The BSA‐coating enabled to markedly improve colloidal stability of Ge NPs in physiological conditions. The mean hydrodynamic diameter of BSA‐Ge NPs remained unchanged in PBS buffer for at least 90 min, while uncoated BSA‐Ge NPs formed large aggregates of 600‐nm size over the same period (Figure 2g). The coated nanoparticles preserved their stability in PBS and DMEM/F12 medium for at least 8 h (Figure S6a,b, Supporting Information). In 1 day, polydispersity of BSA‐Ge nanoparticles steadily increased, while hydrodynamic size distribution start shifting to a greater values in both environments (Figure S6a,b, Supporting Information). This fact can be explained by BSA‐Ge NPs degradation. Indeed, during 24 h of incubation over 67% and 72% of Ge were dissolved in PBS and DMEM/F12, respectively (Figure S7, Supporting Information). Notably, BSA‐Ge particles demonstrate longer half‐lives than uncoated Ge in the same conditions (Figure S2a,c, Supporting Information), estimated to be 6 h in PBS and DMEM/F12. This is not surprising, since modification of nanoparticle surface with hydrophilic moieties is a known approach to slow down the process of oxidation and degradation.^[^ ^13^, ^28^ ^]^

### 2.4 Photothermal Properties of BSA‐Ge Nanoparticles in the NIR‐I Spectral Range

**Figure 3:**
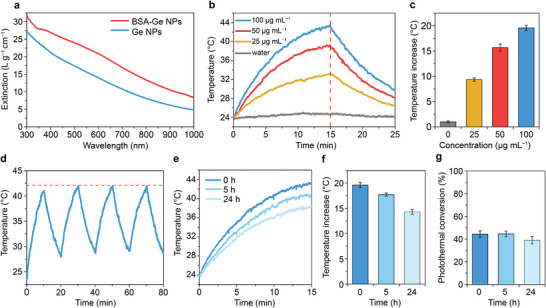
Photothermal properties of BSA‐Ge NPs under NIR‐I irradiation. a) Extinction spectra of uncoated and BSA‐coated Ge NPs normalized to Ge mass. *n* = 1 measurement. b) Photothermal heating/cooling kinetics and c) corresponding peak temperature increments, following 15‐min irradiation of aqueous solutions of different concentrations of BSA‐Ge NPs. d) Photothermal stability of 100 µg mL^−1^ BSA‐Ge NPs during four 20‐min heating/cooling cycles. e) Photothermal 15‐min heating kinetics, f) the corresponding peak temperature increments, and g) the photothermal conversion efficiency of 100 µg mL^−1^ BSA‐Ge NPs, following their incubation in water for different periods. Heating was evaluated using 0.7‐W laser irradiation at 830 nm. Data in (c,f,g) are presented as mean ± SD, *n* = 3 samples in (b‐g).

**Figure** **3**a shows optical extinction spectra of uncoated and BSA‐coated Ge NPs. Albumin‐coated germanium displayed significantly higher optical extinction across the near‐infrared spectral range. We hypothesize that BSA‐Ge NPs can have the larger size due to the dissolution of the small‐sized fraction of particles during the process of BSA‐coating. Theoretical modeling of light extinction by spherical Ge NPs of 50–200 nm size range revealed that particles of 150–200 nm diameter have the highest normalized cross‐section in the red and NIR regions (see details in Note S1, Supporting Information). Note a significant enhancement of the absorbance and scattering in the NIR‐I region was observed for spherical Ge particles larger than 100 nm in diameter (Figure S8, Supporting Information).

Photothermal heating was evaluated in the NIR‐I spectral range using an 830‐nm laser operated at a power of 0.7 W. BSA‐Ge NPs sample in 100 µg mL^−1^ Ge concentration was heated from room temperature (24 °C) to 43.5 °C after 15 min of irradiation (Figure 3b,c, thermal images in Figure S9, Supporting Information). Solution with NPs concentration of 25 µg mL^−1^ was heated by 9.3 °C after 15 min (Figure 3b,c), which was in a range stipulated for medical hyperthermal treatment.^[^ ^32^ ^]^ A comparable temperature increment has been demonstrated using crystalline 4‐nm germanium nanoparticles, although tenfold higher particle concentration was used along with the more favorable illumination conditions: 0.9 W laser power was employed at a wavelength of 770 nm, where the Ge absorption was greater.^[^ ^13^ ^]^ It confirmed beneficial effects of using larger Ge NPs for phototheranostic applications.

Short‐term and long‐term stability of BSA‐Ge NP heating was evaluated during their incubation in water at 100 µg mL^−1^ initial Ge concentration. The sample demonstrated good stability during 1‐h long period of cyclic heating test (Figure 3d), with no apparent photoinduced degradation. However, deteriorations in photothermal generation potency of the BSA‐Ge NPs were revealed during a long‐term period of 1 day (Figure 3e,f). BSA‐Ge NPs was photothermally heated only by 14.3 °C above room temperature after 24‐h incubation in water, which was accompanied by a decrease of the optical density at 830 nm approximately to a half of its initial value (Figure S10, Supporting Information). Note that the photothermal conversion efficiency of BSA‐Ge NPs changed less dramatically in the long‐term stability test, i.e., from 44.4 ± 2.9% at 0 h to 39.1 ± 3.3% after 24 h of incubation (Figure 3g; Figure S11, Supporting Information). Therefore, BSA‐Ge NPs remained highly efficient photothermal converters during the degradation, outperforming such recognized NIR‐photothermal agents as gold and iron oxide nanoparticles in terms of conversion efficiency.^[^ ^33^ ^]^

### 2.5 BSA‐Ge NPs Uptake, Biocompatibility, and NIR‐Phototherapy In Vitro

**Figure 4:**
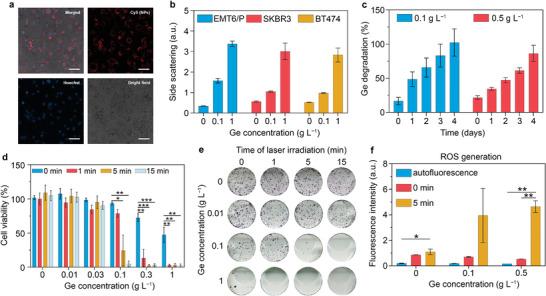
Evaluation of PTT and biodegradation of BSA‐Ge NPs in vitro. a) Representative confocal images of BSA‐Ge NPs binding to EMT6/P cells. BSA‐Ge NPs were labeled with Cy5, cell nuclei were stained with Hoechst. Scale bars – 50 µm. b) Analysis of BSA‐Ge NPs association with EMT6/P, SKBR3, and BT474 cells by side scattering with flow cytometry. c) Ge release kinetics from EMT6/P cells after BSA‐Ge NPs uptake. d,e) MTT (d) and clonogenic assay (e) analyses of EMT6/P cells cytotoxicity dependent on BSA‐Ge NPs concentration and period of 830‐nm laser irradiation. f) Analysis of reactive oxygen species generation following NPs incubation with EMT6/P cells and 830‐nm laser irradiation. ^*^–*p* <0.05, ^**^–*p* <0.01, ^***^–*p* <0.001, one‐way ANOVA with Tukey's post‐hoc test. Data in (b–f) are presented as mean ± SD, *n* = 3 samples.

Photoconverting nanomaterials, such as Ge NPs, can induce necrotic or apoptotic cell death, following their internalization and photoactivation.^[^ ^34^ ^]^ To this aim, we explored BSA‐Ge NP uptake and cytotoxicity in different cancer cells. Confocal microscopy and flow cytometry (**Figure** **4**a,b) were employed to assess the BSA‐Ge NP internalization in mice adenocarcinoma EMT6/P cell line. Accumulation of BSA‐Ge NPs in cells was clearly observable in a bright‐field mode 45‐min post incubation (Figure S12, Supporting Information). Confocal microscopy of NPs labeled with BSA‐conjugated fluorescent Cy5 dye revealed their localization in the endosomes, particles did not stay on the membrane, and did not penetrate into the nucleus (Figure 4a).

High refractive index and large optical scattering of Ge NPs allowed to analyse cell uptake by side scattering (SSC) with flow cytometry, even without fluorescent labelling.^[^ ^35^ ^]^ The SSC histogram of EMT6/P cells was found shifted to greater values in comparison to the untreated cells (Figure S13, Supporting Information), while the median was positively correlated with BSA‐Ge NPs dose (Figure 4b). The side scattering level was similar across the tested cancer cell lines, including human breast adenocarcinoma SKBR3 and human ductal carcinoma BT474 (Figure 4b). The observed high‐level non‐specific binding and uptake of BSA‐Ge NPs to the tested murine and human cancer cells post‐exposure provided ground to assume comparable phototoxicity damage to human tumor cells.

Degradation of BSA‐Ge NPs in cells was quantified by ICP‐MS. The initial uptake of BSA‐Ge NPs at a dose of 100 and 500 µg incubated with 10^6^ of EMT6/P cells were 14 and 62 µg of Ge, respectively. Then we observed gradual release of Ge into the cellular environment over several days (Figure 4c). Cells exposed to BSA‐Ge NPs at 0.1 mg dose demonstrated a burst release of 46% of Ge 1 day post‐incubation, followed by complete particle dissolution within 4 days. The dissolution was slowed down when the greater BSA‐Ge NPs concentration of 0.5 mg was used, with a half of Ge released within the first 2 days, followed by 80% dissolution on day 4. These results indicated a likely case scenario of rapid Ge nanoparticle degradation in acidic endosomal and lysosomal environments in vivo.

Next, we investigated cytotoxicity and photothermal effect of BSA‐Ge NPs under 830‐nm laser irradiation using EMT6/P cell line (Figure 4d). The laser treatment at 0.7 W power in the absence of the nanoparticles did not significantly impact metabolic activity of cells. Also, without irradiation, BSA‐Ge NPs showed high biocompatibility with IC_50_ of 0.86 mg mL^−1^, calculated from the MTT viability assay. The cytotoxicity of BSA‐Ge NPs increased markedly under NIR irradiation. The IC_50_ levels for 1, 5, and 15‐min laser exposure were 0.16, 0.07, and 0.05 mg mL^−1^, respectively. The viability of cancer cells was below 5% at a BSA‐Ge NP dose of 0.1, 0.3, and 1 mg mL^−1^ under the respective laser exposures of 15, 5, and 1‐min (Figure 4d). Analysis of the proliferative activity of the cells using a clonogenic assay confirmed a drastic photothermal effect induced by BSA‐Ge NPs treatment (Figure 4e). The complete tumor cells treatment with no detected formed colonies was observed for 0.1 g L^−1^ Ge concentration under 15‐min laser irradiation.

While a necrotic pathway due to the cell overheating was hypothesized to be the main contributor to the cell death during the therapy, an oxidative stress induction via photodynamic effect can synergize with this pathway.^[^ ^36^ ^]^ The production of reactive oxygen species (ROS) during the photothermal treatment of cells was assayed by a fluorogenic carboxy‐H2DCFDA probe and flow cytometry showing a strong enhancement of the cell fluorescence as a result of the NIR irradiation in the presence of BSA‐Ge NPs (Figure 4f). We observed a (5.6 ± 3.0) and (8.6 ± 0.9)‐fold increase of the probe fluorescence upon cell exposure to 0.1 and 0.5 mg mL^−1^ of Ge in comparison with untreated cells. Notably, neither separate NIR irradiation nor BSA‐Ge NPs exposure resulted in ROS production (Figure 4f).

The photocatalytic activity of BSA‐Ge NPs under NIR‐I irradiation was confirmed by evaluating degradation of Rhodamine B fluorescent dye, which is susceptible to oxidation (Figure S14, Supporting Information).^[^ ^37^ ^]^ The optical absorbance of 50‐µm Rhodamine B incubated with 1 g L^−1^ Ge NPs steadily decreased under 1‐W laser exposure, reaching 66% of the dye degradation after 30 min. In comparison, only 24% of the dye was eliminated if Rhodamine B was incubated with Ge NPs under dark condition, probably due to the dye adsorption to nanoparticle surface and medium acidification. No optical absorbance decrease over 30‐min period was detected in case of the solely 1‐W laser irradiation of Rhodamine B or dye incubation at 60 °C, so that the effects of heating and laser exposure on the dye degradation were ruled out.

The photoexcitation‐induced oxidative stress has been previously observed in other germanium‐based nanomaterials.^[^ ^38^ ^]^ Our results suggested that a combined action of the photothermal and photodynamic effects can ensure the effective destruction of resistant tumor cells during phototherapy with BSA‐Ge NPs.

### 2.6 Pharmacokinetics of BSA‐Ge Nanoparticles

The blood circulation and biodistribution profile of BSA‐Ge NPs were measured in BALB/c mice. To this aim, nanoparticles were injected intravenously at a dose of 0.25 mg of Ge, and blood samples were collected at several time points. Three animals were sacrificed after 3 h; 2, 7, 14, and 28 days, their main organs were collected to assay the concentration of Ge by the mass‐spectrometry. The kinetics of the nanoparticle circulation in the blood demonstrated a monoexponential decay, with a half‐life of *t_1/2_* = 110 ± 20 s (Figure S15, Supporting Information). This circulation time was typical for 100‐nm non‐PEGylated particles administered at a low dose.^[^ ^39^ ^]^

**Figure 5:**
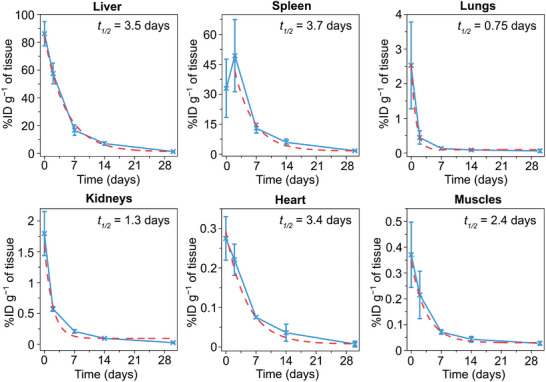
Biodistribution and degradation of BSA‐Ge NPs in vivo. BSA‐Ge NPs were injected intravenously in BALB/c mice and Ge concentration in major organs was analysed by ICP‐MS. (*n* = 3 animals for each point). Red lines show monoexponential fitting of the experimental data.

Biodistribution and biodegradation properties of BSA‐Ge NPs are shown in **Figure** **5**. After the elimination from the bloodstream, most of Ge accumulated in the liver and spleen in concentrations of 86 ± 9%ID g^−1^ and 33 ± 15%ID g^−1^, respectively. These two organs are part of mononuclear phagocyte system and contain a number of macrophages, recognizing and removing exogenous materials from the circulation.^[^ ^40^ ^]^ The Ge concentration in the liver gradually decreased with a half‐life of *t*_1/2_ = 3.5 ± 0.3 days. In the spleen, the Ge concentration raised to 49 ± 18%ID g^−1^ on Day 2, followed by a gradual decrease with *t*_1/2_ = 3.7 ± 1.5 days. Presumably, the spleen served as a metabolic depot for Ge compounds in a short‐term period, which was consistent with previous observations of Germanium fluctuations in the murine spleen within 2 days after an injection of sodium germanate.^[^ ^41^ ^]^ In the lungs and kidneys, the highest Ge uptake was reached 3 h post‐injection with concentrations of 2.5 ± 1.3%ID g^−1^ and 1.8 ± 0.4%ID g^−1^, respectively. Ge was eliminated faster in these organs compared to the liver with half‐lives of 0.75 ± 0.14 days and 1.3 ± 0.3 days in the lungs and kidneys, respectively. The uptake of Ge nanoparticles by the heart and muscles was at low levels (≈0.3 %ID g^−1^), gradually decreasing with time (Figure 5). The Ge accumulation in these organs was significantly lower compared to the other forms of inorganic Germanium, such as Na_2_GeO_3_,^[^ ^41^ ^]^ GeO_2_,^[^ ^42^ ^]^ and GeCl_4_,^[^ ^43^ ^]^ which were predominantly accumulated in the kidneys and lungs. A whole‐body clearance of BSA‐Ge nanoparticles calculated from the major organs was characterized by a half‐life of 3.5 ± 0.4 days (Figure S16, Supporting Information), which confirmed rapid biodegradation behavior of Ge in vivo.

### 2.7 NIR‐Photothermal Therapy In Vivo

**Figure 6:**
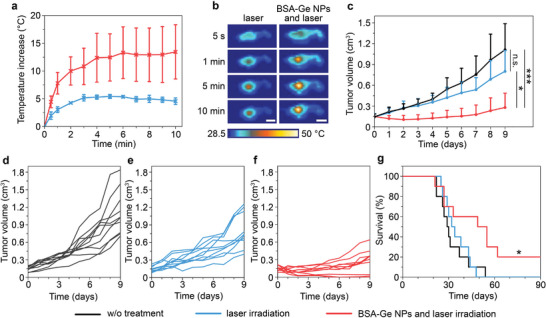
NIR‐I photothermal treatment in vivo. a) Kinetics of the temperature rise in the tumor during 10 min of heating using 830‐nm laser exposure. *n* = 4 animals. b) Representative time‐lapse thermographic images of untreated and BSA‐Ge NPs‐treated mice during the PTT laser exposure. Scale bars – 2 cm. c) The tumor volume growth kinetics after the treatment. *n* = 10 animals in each group. d‐f) Tumor growth kinetics of individual animals: d) without treatment, e) after laser treatment, f) after BSA‐Ge NPs and laser treatment, *n* = 10 animals in each group. g) Kaplan‐Meier plot of animal survival after the treatment, *n* = 10 animals in each group. In a,c) data presented as mean ± SD. Statistical analysis was performed by ANOVA with Tukey's post‐hoc test c) or log‐rank test g). n.s. −*p* >0.05, ^*^−*p* <0.05, ^***^−*p* <0.001.

BSA‐coated Ge NPs were evaluated for photothermal treatment of EMT6/P adenocarcinoma in BALB/c mice. Tumor cells were inoculated subcutaneously in the flank region and monitored until the tumor volume reached 150 mm^3^. Then, nanoparticles at a 1.2‐mg Ge dose were intravenously administrated in mice, and tumor region was irradiated by 830‐nm laser in 20 min. The animals were divided into three groups: animals received no treatment (“w/o treatment”); exposed to 2 W cm^−2^ laser irradiation for 10 min (“laser” group), treated by the same irradiation after a nanoparticle injection (“BSA‐Ge NPs + laser”). The temperature of the tumor lesion was increased by 14 and 5 °C immediately after the PTT treatment in the tested and control groups, respectively, as measured by an infrared camera (**Figure** **6**a,b).

The photothermal treatment with BSA‐Ge NPs showed significant therapeutic benefits (Figure 6), while no severe toxicity was found associated with the decrease of the animal's body weight (Figure S17, Supporting Information). We observed a decrease of the mean tumor volume 2 days post‐treatment from 148 ± 43 to 106 ± 61 mm^3^ in “BSA‐Ge NPs + laser” group, while the tumor volume increased to 265 ± 67 and 277 ± 95 mm^3^ in the group received only laser treatment and animals without treatment, respectively (Figure 6c). In the following period, “BSA‐Ge NPs + laser” group exhibited the slower tumor growth rate than the other animals. On Day 9, the average tumor volume of these animals reached 280 ± 206 mm^3^, i.e., less than twofold of its original volume, while the average tumor volume increased 5.5‐fold to 0.8 cm^3^ and 7.6‐fold to 1.1 cm^3^ in the laser‐treated and non‐treated groups, respectively. We emphasize that several animals treated with BSA‐Ge NPs and NIR laser displayed complete tumor eradication (Figure 6d–f). Besides, a significant prolongation of the animal survival in the post‐treatment period was observed after the photothermal treatment (Figure 6g). Median lifespans of the animals were 30, 34, and 54 days for the “w/o treatment”, “laser”, and “BSA‐Ge NPs + laser” groups, respectively. The PTT treatment results demonstrated the promise of BSA‐Ge NPs as a photothermal nanomedicine at in vivo level and prompted further investigation of its applicability to photoacoustic imaging.

### 2.8 Photoacoustic Imaging

To evaluate the feasibility of utilizing Ge nanoparticles for photoacoustic imaging, we examined their photoacoustic properties. PAI relies on photoinduced heating of light‐absorbing agents resulting in generation of ultrasound waves via thermoelastic transduction. The photoacoustic properties of Ge NPs were measured using a TriTom tomograph under 830‐nm pulsed laser excitation.

**Figure 7:**
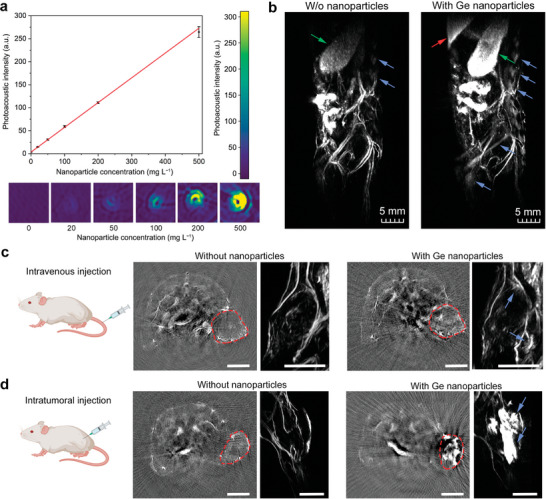
Photoacoustic imaging of BSA‐Ge NPs. a) Dependence of photoacoustic signal intensity on Ge NP concentration in capillaries. Red line shows linear fitting. n = 3 samples. b) Whole body PAI of a mouse before and after intravenous injection of BSA‐Ge NPs. Red arrow points to the liver, green arrow – to the spleen, blue arrows – to the bones. c,d) PAI of EMT6/P subcutaneous tumors before and after intravenous (c) or intratumoral (d) administration of BSA‐Ge NPs. Each image shows axial cross‐section (left) and 3D reconstruction of the tumor region (right). Red boundaries indicate tumors. Scale bars – 5 mm. Schemes were created with BioRender.com.

A photoacoustic signal acquired from thin capillaries filled with aqueous solutions of Ge NPs displayed linear dependence versus material concentration in a range of 20–500 µg mL^−1^ (**Figure** **7**a). The lowest detection limit was estimated to be 2.3 µg mL^−1^ (confidence level 95%). Moreover, Ge NPs in 20 µg mL^−1^ concentration provided a 4.2‐fold contrast enhancement in comparison to that of distilled water.

Then we carried out PAI of BALB/c mice. An apparent photoacoustic contrast of the blood vessels and spleen was notable in the absence of BSA‐Ge NPs and attributed to the strong light absorption by hemoglobin.^[^ ^44^ ^]^ After an intravenous administration of BSA‐Ge NPs, we observed an enhanced contrast in the liver and spleen, as anticipated in virtue of significant accumulation of the NPs in these organs (Figure 7b). In addition, the injected nanoparticles rendered the vertebral column and femur bones high photoacoustic contrast, as compared to almost undetectable skeletal structures of the untreated mice (blue arrows, Figure 7b). The observed tropism of Ge NPs to the bones can be explained by the particle‐capturing activity of bone marrow phagocytes and osteoclasts in the tissue. Moreover, bone tropism is well‐established for molecular forms of germanium, but still lacks proper biological explanation.^[^ ^45^ ^]^ This observation points to opportunities for photoacoustic imaging and diagnostics of bone morphology and relevant diseases with nanoformulated germanium.

Then we evaluated PAI of EMT6/P tumors, subcutaneously inoculated in BALB/c mice (Figure 7c,d). Bright spontaneous flashes in the tumor periphery were observed in the vicinity of the tumor vessels 3‐h post intravenous administration of 1.2 mg of BSA‐Ge NPs (blue arrows, Figure 7c). A photoacoustic contrast of the tumor was also enhanced, as seen in the axial cross‐sections of the animal. Note that the PA contrast was non‐uniform across the tumor, which is common situation for solid tumors, where contrast agent diffusion is hampered by the dense extracellular matrix presence.^[^ ^46^ ^]^ To enhance tumor contrast, in a separate set of experiments, we injected BSA‐Ge NPs of the same dosage intratumorally, followed by PAI 5‐min post‐administration (Figure 7d). After the injection, the tumor region displayed the highest contrast in the organism, providing better PA enhancement than that of hemoglobin abundant in the blood and other internal structures.

To push the frontiers of the emerging PAI technique, a repertoire of contrast agents suitable for in vivo imaging must be expanded. In this study we scrutinized suitability of Ge NPs as the contrast agent for PAI at 830‐nm wavelength. An extinction spectrum of BSA‐Ge NPs displayed a monotonic decrease in the 650–950 nm spectral range known as NIR‐I biological tissue transparency window (Figure 3a). The mass extinction measured as high as 19 cm^−1^ g L^−1^ at 650 nm dropped by ca 50% to the value of 9.5 cm^−1^ g L^−1^ at 950 nm. This showed potential of nanosized germanium material as a high‐quality photoacoustic imaging agent in the broad spectral range.

### 2.9 Analysis of Toxicity of BSA‐Ge Nanoparticles at a Therapeutic Dose

Systemic administration of nanomaterials always raises concerns with regard to potential toxicological hazards and must be evaluated in terms of their cytotoxicity and tissue inflammatory response.^[^ ^47^ ^]^ To this aim, we investigated Ge NPs toxicological properties by measuring their interaction with red blood cells (RBCs), analyzing relevant blood biochemistry markers, and performing histopathological evaluation of the major organs.

**Figure 8:**
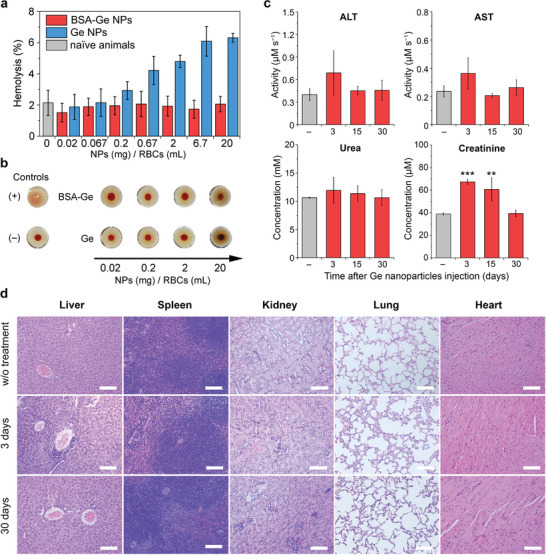
Toxicology of BSA‐Ge nanoparticles after intravenous administration. a) Hemolytic activity of uncoated and BSA‐coated Ge NPs versus concentration. *n* = 10 samples for naïve animals and *n* = 3 for particle‐treated groups. b) Representative images of the hemagglutination test after RBC incubation with Ge or BSA‐Ge NPs. Non‐agglutinated RBCs sediment into the dot in the center of a well. c) Activity or concentration of the blood biochemical markers: alanine transaminase (ALT), aspartate transaminase (AST), blood urea and creatinine. Grey bars show untreated animals. *n* = 4 samples per each data point. d) Histological images of the major organs of untreated mice and animals after BSA‐Ge NPs treatment on Days 3 and 30. Scale bars – 1 mm. Data in (a, c) are presented as mean ± SD. ^**^−*p* <0.01, ^***^−*p* <0.001, ANOVA with Tukey's post‐hoc test.

Blood hemolysis and hemagglutination were evaluated using murine red blood cells. Uncoated Ge nanoparticles showed dose‐dependent hemolytic activity, with RBC lysis observed at Ge NP concentrations greater than 0.2 mg mL^−1^ (**Figure** **8**a). At the same time, BSA‐coated Ge NPs did not cause RBC damage even in the 100‐fold higher concentrations (20 mg mL^−1^), and hemolysis level was comparable to spontaneous lysis in naïve animals (Figure 8a). Hemagglutination test was carried out by employing a standard assay with U‐shaped wells, where sedimentation of the aggregated RBCs formed a diffuse disk, while dispersed RBCs formed a dot on the bottom of the well. Ge and BSA‐Ge NPs at concentrations of 0.02–20 mg mL^−1^ were incubated with RBCs for 1 h in wells. No agglutination of RBCs was observed for both uncoated and BSA‐coated Ge NPs in the tested concentration range (Figure 8b). These tests indicated that BSA coating of Ge NPs prevented RBC damage. Therefore, only BSA‐coated Ge NPs were used for toxicity tests in our further in vivo studies.

Blood biochemical analysis was performed on Days 3, 15, and 30 post intravenous administration of 1.2‐mg BSA‐Ge NPs in BALB/c mice. We analyzed standard stress markers for the liver and kidney, including activity of alanine transaminase (ALT), aspartate transaminase (AST), as well as creatinine and blood urea levels. These organs were responsible for the renal and biliary excretion of BSA‐Ge NPs during their degradation and were likely to be impacted. No significant alterations of ALT and AST activity of BSA‐Ge NPs were revealed up to Day 30 post‐injection, indicating no acute or chronic damage to the liver function (Figure 8c). Also, the blood urea level remained normal within the observation period (Figure 8c). The concentration of creatinine was elevated on Days 3 and 15, then returned to normal values on Day 30. This increase of the enzyme concentration was comparable to the levels observed in mice treated with other nanotherapeutics, including clinically approved Caelyx.^[^ ^48^ ^]^

Histopathological evaluation showed no changes in the heart, spleen, and lung tissues, as compared to naïve mice. On Day 3 post‐injection, we observed a 10% increase in the number of Kupffer cells in the liver, which is a common acute reaction to the injection of nanoparticles.^[^ ^49^ ^]^ The liver histology displayed normal tissue structure without dystrophic changes on Day 3, although signs of mild inflammation were observed. Indicators of the inflammation and cellular composition of the liver returned to normal values on Day 30. The kidney histology displayed normal organ macro‐ and microstructure. No signs of acute kidney tissue inflammation were found in comparison with naïve animals on Day 3, except for lymphoid infiltration of the glomeruli, mild oedema, as well as increased blood filling of the vessels. Besides, granular vacuolated cytoplasm of the glomerular epithelial cells was observed. These changes returned to normal levels on Day 30 and indicated an absence of chronic toxicity caused by the administered Ge nanomaterial. We infer that the observed acute, yet reversible, stress to the kidney tissue and function was due to the massive BSA‐Ge NP dissolution within the first several days after administration, followed by clearance of their degradation products via the renal filtration.

## 3 Discussion

We demonstrated the promise of Ge NPs as a multifunctional photothermal agent with a low‐toxic degradation pathway suitable for therapeutic and diagnostic applications. Although attractive photothermal properties of Ge have been reported,^[^ ^13^ ^]^ biocompatibility of Ge material remained largely unexplored. We addressed this knowledge gap, paving the way for Ge NPs‐based PTT and PAI applications, especially in the biological tissue transparency window.

Our method of pulsed femtosecond laser ablation in liquids yielded spherical nanoparticles with 70‐nm mean size characterized by preserved partial crystallinity and endowed hydrophilicity. The nanoparticle size can be optimized during the procedure of the laser ablation per se or augmented by subsequent laser fragmentation.^[^ ^50^, ^51^ ^]^ In contrast, popular chemical methods of Ge NP synthesis by reduction of Germanium salts or thermal decomposition of organogermane precursors usually yielded sub‐20‐nm Ge particles by initial reaction, while the larger particles were usually aggregated or coalesced.^[^ ^52^ ^]^ Top‐down Ge NP production approaches, including high‐energy ball milling yielded sub‐micron Ge NPs with polydisperse size distribution characterized by irregular angled shapes.^[^ ^14^ ^]^ Since large optical extinction of photothermal nanomaterials is pivotal for photoacoustic and photothermal applications, size tunability of Ge NP featuring strong Mie resonances in the NIR‐I range (Note S1, Supporting Information) is important and comfortably furnished by the laser ablation method. Nanoparticles with sizes smaller than 100 nm in diameter appeared unfavorable for phototheranostic applications. Indeed, the BSA‐coating of as‐synthesized Ge NPs shifted the mean‐diameter of the original particle size distribution (toward >100 nm) and markedly enhanced the mass optical extinction (Figure 3a).

Since Germanium belongs to group IV materials, its chemical properties are expected to be similar to that of silicon. Silicon NPs have been broadly used in gene, drug delivery, and photoluminescent imaging, largely owing to their proven biodegradability.^[^ ^15^ ^]^ This broad application scope as well as global semiconductor industry demands have led to the development of numerous NP surface modification protocols primarily based on the Stöber reaction of silanes hydrolysis.^[^ ^53^ ^]^ We anticipate the surface modification of Ge particles will be greatly facilitated by adaptation of these protocols and further involvement of coating with stealth polymers and conjugation with targeting moieties. One needs to be cautious considering formation of solid silicon oxide layer on the particle surface can slow down the degradation rate and compromise the biodegradability.

Germanium and silicon share the same degradation pathway via an oxidation and subsequent hydrolysis reactions.^[^ ^18^ ^]^ Nevertheless, the dissolution kinetics of laser‐ablated Ge NPs was faster than that of solid Si NPs,^[^ ^54^ ^]^ favoring Ge for biomedical applications. In this study, we performed first quantitative evaluation of Ge NP degradation in mammals using ICP‐MS, a state‐of‐play in the analytical methodology, which analyzed elimination of the Ge degradation products from the tissues. A whole‐body clearance of BSA‐Ge NPs was characterized by a half‐life of *t*_1/2_ = 3.5 ± 0.4 days. This clearance rate was considerably faster compared to that of other nanoparticles widely used in biomedicine, such as TiO_2_ (*t*_1/2_ ≈10 days),^[^ ^55^ ^]^ polymer‐stabilized iron oxides (*t*_1/2_ >15 days),^[^ ^9^ ^]^ gold (*t*_1/2_ = 73 days),^[^ ^56^ ^]^ and soluble silica NPs (*t*_1/2_ ≈30 days).^[^ ^57^ ^]^ The short half‐life of BSA‐Ge nanoparticles in the organism alleviated risks of chronic inflammation caused by the long‐term particle retention. This risk is well‐known for non‐biodegradable noble metal nanoparticles^[^ ^58^ ^]^ and carbon nanomaterials.^[^ ^59^ ^]^ Moreover, a Ge NP dissolution product, tetravalent germanium, is known to have cytotoxicity lower ^[^ ^60^ ^]^ compared to the other multivalent metal ions,^[^ ^61^ ^]^ so the catabolism of Ge NPs is likely to exert minimal toxic stress on the organism. It has been confirmed in our study and elsewhere,^[^ ^14^ ^]^ with no chronic toxicity observed in the tissues.

Molar extinction coefficients of Ge NPs appeared superior in comparison with the other optical absorbers. To compare Ge nanomaterial with the other reported light‐absorbing nanoparticles used for PTT and PAI, we collated in Note S2 (Supporting Information) their optical properties evaluated at a popular NIR‐I wavelength of 808 nm. Molar extinction of unmodified Ge was calculated as 0.57 cm^−1^ mm ^−1^, which was comparable to the state‐of‐play nanomaterial, black‐phosphorus quantum dots (0.46 cm^−1^ mm ^−1^),^[^ ^62^ ^]^ trailing particles composed of heavier inorganic compounds, such as MXenes, and noble metals. However, comparing the extinction per particle, Ge NPs are at the top of the list of nanomaterials used in phototherapy (Table S1, Supporting Information). The extinction per Ge nanoparticle of 67 nm in diameter was calculated to be 4 × 10^9^ cm^−1^ M^−1^ comparable to the best PTT performers like red‐shifted plasmon Au nanorods (5.6 × 10^9^ cm^−1^ M^−1^)^[^ ^63^ ^]^ and Pd nanosheets (4.1 × 10^9^ cm^−1^ M^−1^).^[^ ^64^ ^]^ Such high NIR‐light extinction of these noble nanomaterials enabled their successful phototheranostic applications reported to date.^[^ ^65^ ^]^ However, since these metal particles are not biodegradable, their application scope, including clinical applications, is limited. Among biodegradable nanomaterials suitable for PAI and PTT, Ge NPs seem to be superior, with the molar extinction threefold greater than that of the runner‐up, Prussian‐blue nanoparticles (1.1 × 10^9^ cm^−1^M^−1^). It is important to note that these calculations were performed for uncoated as‐synthesized Ge NPs. BSA‐coated Ge NPs featured 1.6‐fold higher mass extinction coefficients (Figure 3a) due to partial dissolution of the smallest fraction during the coating. Therefore, the promise of our spheroidal BSA‐Ge nanoparticles for phototheranostic applications is obvious.

Several other issues need further optimization and left behind this study. First, the size of particles reported here seems sub‐optimal. Particles larger 200 nm are rarely used for intravenous injection due to the enhanced sequestration by macrophages. On the other hand, spherical Ge NPs of 150–200 nm in diameter were calculated to exhibit optical extinction Mie resonances in red and NIR spectral ranges (Note S1, Supporting Information). Obviously, this will enable high‐contrast photoacoustic imaging and higher efficacy of PTT. Based on our theoretical modeling results, we inferred that the contribution of particles with the diameter larger than 100 nm was dominant to our reported treatment outcomes. Mean size and polydispersity of Ge NP colloids must be optimized for better performance in specific in vivo scenarios.

Second, in this study, we used solid Ge nanoparticles. As such, these are poorly suited to deliver drugs due to limited numbers of surface docking moieties. Porous Ge nano‐ or submicron particles represent a promising material to realize both PAI/PTT and drug delivery in one agent. Drug release can be thermally triggered by laser radiation or a high‐power radiofrequency source. The development of porous Ge nanoparticles sized 100–200 nm represents a viable alternative to widespread silicon‐based drug delivery vehicles due to similar biocompatibility but faster dissolution rate of Ge NPs. To this aim, one needs to consider approaches alternative to the reported pulsed laser ablation in liquids, unsuitable for production of porous nanomaterials.

## 4 Conclusion

In conclusion, we scrutinized an emerging photothermal converter, Germanium nanomaterial, for photoacoustic imaging and photothermal therapy applications, with an emphasis on its biocompatibility in vivo. We synthesized crystalline Ge nanoparticles by pulsed laser ablation in liquids, followed by albumin surface coating to improve compatibility in biological systems. Biodegradability of Ge NPs was demonstrated by rapid dissolution in salt buffers through an oxidation mechanism, which was preserved in tumor cells in vitro, and in normal tissues in mice with a half‐life of 3.5 days. Low toxicity of Ge NPs was confirmed in cells and in vivo, showing only minor and reversible changes in the kidneys with negligible hematotoxicity. To demonstrate potential of our Ge NPs for photothermal therapy, we treated EMT6/P adenocarcinoma in mice with deeply penetrating near‐infrared radiation at 830 nm and achieved a 3.9‐fold reduction of the tumor growth and prolonged animal survival. A promise of Ge NPs for photoacoustic imaging was confirmed by rendering apparent photoacoustic contrast to the bones and tumors following intravenous or intratumoral NP administrations. Taken together, the demonstrated safety profile of biodegradable Ge nanomaterial makes it an outstanding photothermal agent for advanced PAI/PTT theranostics, with prospects of drug delivery and photoluminescent imaging applications.

## 5 Experimental Section

### Materials

Sigma–Aldrich (USA): bovine serum albumin (BSA); acetone; nitric acid; tween 80; 3‐(4,5‐dimethyl‐2‐thiazolyl)−2,5‐diphenyl‐2H‐tetrazolium·bromide (MTT); dimethylsulfoxide (DMSO); Hoechst 33 342; crystal violet; formaldehyde; Rhodamine B. Virbac (France): zoletil. Bioveta (Czech Republic): rometar. Sintez (Russia): heparin. BioXcell (USA): rat anti‐mouse TER 119 antibodies. Jackson ImmunoResearch (USA): goat anti‐rat IgG antibody. Gibco (UK): DMEM/F12 with GlutaMAX and HEPES, without phenol red; heat‐inactivated fetal bovine serum (FBS); antibiotic‐antimycotic solution; Versene solution. Invitrogen (USA): carboxy‐H2DCFDA. Lumiprobe (Russia): Cyanine5 NHS‐ester.

### Cells

EMT6/P cells of mouse mammary carcinoma were obtained from ECACC collection. BT474 cells of ductal carcinoma of human mammary gland, SKBR3 cells of adenocarcinoma of human mammary gland were obtained from ATCC collection. Cells were cultured in DMEM/F12 medium with GlutaMAX, supplemented with 10% FBS and antibiotic‐antimycotic solution in culture flasks, under a humidified atmosphere at 5% CO_2_ and 37 °C.

### Animals and Ethics Approval

Female BALB/c (10–14 weeks old, 18–22 g) mice were used in the study. Animals were obtained from Pushchino Animal Facility (Pushchino, Russia) and maintained in a vivarium at Shemyakin‐Ovchinnikov Institute of Bioorganic Chemistry. All experimental procedures were approved by the Institutional Animal Care and Use Committee (protocol № 240) of Shemyakin‐Ovchinnikov Institute of Bioorganic Chemistry. The mice were housed under a 12‐h light/dark cycle with free access to food and water. Animals were anesthetized with an intraperitoneal injection of mixture of Zoletil and Rometar in a dose 40 and 1.6 mg kg^−1^, respectively, prior to any nanoparticle injections.

### Synthesis of Ge Nanoparticles

The synthesis of Ge nanoparticles was carried out via femtosecond pulsed laser ablation of germanium wafer in acetone (Figure S1, Supporting Information). The wafer was positioned vertically along a side wall of the glass cuvette so that the distance between the target and opposite wall of cuvette was 4 mm. A radiation beam of 3 mm diameter generated by a Yb:KGW laser (1030 nm wavelength, 270 fs pulse duration, 30 µJ pulse energy, 100 kHz repetition rate; TETA 10, Avesta, Russia) was focused on the surface of the target by a F‐theta lens (f = 100 mm). Using a galvanometer optical scanner, laser beam was set to scan a 5 × 10 mm area of target surface at 4000 mm s^−1^ speed for 60 min. After the ablation procedure, the obtained germanium nanoparticles were purified by centrifugation in acetone (10 min, 8000 g). To remove Ge microspheres, which can be formed during the synthesis, nanoparticles were centrifuged at 100 g for 2 min and supernatant was used for further experiments.

### Coating of Ge Nanoparticles

Ge particles in acetone were washed three times with distilled water by centrifugation (10 min, 15 000 g) to remove acetone residues. Then, particles at 1 g L^−1^ concentration were incubated in 5% BSA solution for 30 min under continuous stirring at room temperature. The non‐bound proteins were removed by centrifugation (10 min, 15 000 g) and particles were washed by distilled water three times. Mass of undissolved Ge after the coating was determined by ICP‐MS. The amount of adsorbed protein after the coating procedure was measured by Bradford assay using Infinite M1000 PRO spectrophotometer at 595 nm wavelength.

### Nanoparticle Characterization

Nanoparticle size and morphology were analyzed by high‐resolution transmission electron microscopy with Titan Themis Z microscope (Thermo Fisher Scientific, Netherlands) coupled with Super‐X energy dispersive X‐ray detector. Degradation of nanoparticles was analyzed by scanning electron microscopy using Tescan MAIA 3 device (Tescan, Czech Republic) and X‐act energy dispersive detector (Oxford Instruments, High Wycombe, UK) at 25 kV accelerating voltage.

Raman spectra were measured by a Confotec MR350 confocal micro‐Raman spectrometer (SOL instruments, Belarus) under excitation with 633‐nm laser. UV–vis extinction spectra were recorded by Infinite M1000 PRO spectrophotometer (Tecan, Austria).

The hydrodynamic diameter and ζ‐potential of nanoparticles were measured by dynamic and electrophoretic light scattering with a Zetasizer Nano ZS device (Malvern Instruments, UK). Number‐size distribution was used for hydrodynamic size analysis. To measure ζ‐potential nanoparticles were incubated in 10 mm NaCl for 15 min, mobility was derived with Smoluchowski approximation.

### Study of Photothermal Properties of BSA‐Ge NPs

Photothermal heating of BSA‐Ge NPs was evaluated after irradiation of solution with 830‐nm laser at 0.7 W power. BSA‐Ge NPs were dispersed in distilled water and thoroughly sonicated. Cuvette with 2 mL of suspension was irradiated with laser for 15 min for concentration and long‐term stability tests, or for 10 min in cyclic test. After these periods, solutions were naturally cooled. Temperature in liquid volume during heating and cooling was monitored by an infrared thermographic camera FLIR C3 (FLIR Systems Inc., USA) and analyzed by FLIR Tools software. Surrounding temperature during experiments was kept at 24 ± 0.5 °C. To determine photothermal conversion efficiency, Roper's model ^[^ ^66^ ^]^ of heat transfer was used (Equation 1).

(1) $$\eta \left(\%\right)=\;\frac{mC\left(\Delta T_{\max}^{\mathrm{NP}}-\Delta T_{\max}^{\mathrm{water}}\right)}{\tau_{c}I\left(1-10^{-A_{830}}\right)}$$

where m and C are mass and heat capacity of solution, respectively; $\Delta T_{max}^{NP}$ and $\Delta T_{max}^{water}$ are elevations of temperature for nanoparticles and water, respectively; τ_*c*_ is cooling temperature coefficient; *I* is irradiation power; *A*_830_ is extinction of nanoparticle solution for 1 cm path length in water. τ_*c*_ was determined from cooling kinetics *T*(*t*) of solution as a slope of the negative logarithm of transfer driving force Θ versus time (Equations (2), (3)).

(2) $$\tau_{c}=\frac{-Ln\left(\Theta \right)}{t}$$

(3) $$\Theta \;=\frac{T\left(t\right)-T_{\max}^{\mathrm{NP}}}{T_{\max}^{\mathrm{NP}}-T_{s}}$$

### Mass‐Spectrometry Study of Ge NP Degradation

Ge NPs were dispersed in distilled water, buffers, or cell medium at 0.1 g L^−1^ concentration. At certain time points, aliquots of 120 µL volume were taken and centrifuged for 10 min at 15 000 g. Then, supernatant and residue were separated, 120 µL of 70% nitric acid were added to each sample, and solutions were incubated for 15 min at 80 °C. Then samples were diluted to 10% nitric acid concentration with distilled water or salt buffer. The concentration of Ge was measured by inductively coupled plasma mass spectrometry with a NexION2000 (Perkin Elmer, USA). The mean concentration, determined over ^70^Ge, ^72^Ge, ^73^Ge, ^74^Ge, and ^76^Ge isotopes was used for analysis.

The following buffers were used for the measurement of nanoparticle degradation:

- Phosphate buffered saline (PBS, 100 mM, pH 7.4), composition: 90 mm of NaCl, 1.8 mm of KCl, 7 mm of Na_2_HPO_4,_ and 1.2 mM of KH_2_PO_4_.
- Simulated body fluid (SBF, 246 mm, pH 7.4), composition: 143 mm of NaCl, 3 mm of KCl, 4.2 mm of NaHCO_3_, 2.6 mM of CaCl_2_, 1.5 mM of MgCl_2_, 0.5 mm of Na_2_SO_4_, 1 mm of K_2_HPO_4_, 50.5 mm of NH_2_C(CH_2_OH)_3_, 39 mm of HCl.
- Citric buffer (100 mm, pH 4.5), composition: 53.6 mm of citric acid, 56.4 mm of trisodium citrate.
- Simulated lysosomal fluid (SLF, pH 4.5), composition: 108 mM of citric acid, 0.5 mM of MgCl_2_, 55 mm of NaCl, 0.5 mm of Na_2_HPO_4_, 0.27 mm of Na_2_SO_4_, 150 mm of NaOH, 0.9 mm of CaCl_2_, 0.3 mm of trisodium citrate, 0.8 mm of glycine, 4.6 mm of sodium tartrate, 0.8 mm of sodium lactate, 0.8 mm of sodium pyruvate.
- Complete Dulbecco's Modified Eagle Medium (DMEM/F12) with GlutaMAX and HEPES (pH 7.4).
- Complete DMEM/F12 supplemented with 10% of fetal bovine serum.

### Study of RBC‐Related Toxicity

Mouse blood samples was taken by puncture of retro‐orbital sinus and collected in anti‐coagulating tubes. Red blood cells were washed twice with PBS via centrifugation (300 g, 5 min) and were resuspended in PBS or 1% BSA in PBS for hemolysis and hemagglutination assays, respectively. Uncoated Ge NPs were washed 4 times with water to remove acetone from particle surface; BSA‐Ge NPs were used immediately after coating.

In hemolysis assay, Ge NPs were added to RBC suspension, containing 5% hematocrit, and incubated at room temperature for 30 min under mild stirring. Then, cells were centrifuged at 300 g, 5 min, and supernatant samples were collected and centrifuged at 15 000 g, 5 min. The optical absorption of samples was measured at 540 nm using Infinite M1000 PRO microplate reader. Samples of RBCs diluted in 1% Tween 80 or PBS were used as positive and negative hemolysis controls, respectively.

A hemagglutination study was performed in 96‐well U‐shaped plates. Ge NPs were incubated with 100 µL 1% hematocrit RBC suspension at room temperature for 1 h. RBCs in 1% BSA solution in PBS was used as negative control. For a positive control, RBCs were incubated for 10 min with rat anti‐mouse RBC antibody (TER‐119, 50 µg mL^−1^), washed with PBS to remove unbound protein, and were incubated with secondary goat anti‐rat antibody (75 µg mL^−1^). Agglutination of RBCs was indicated by formation of a diffuse film on the bottom of the well, while non‐agglutinated RBCs sedimented in the center of the well.

### Measurement of Nanoparticle Binding to Cancer Cells

EMT6/P, BT474, or SKBR3 cells at a concentration 10^6^ cells mL^−1^ were incubated with BSA‐Ge nanoparticles for 45 min at 37 °C in phenol red‐free DMEM/F12 medium containing 10% FBS. Next, unbound NPs were removed by centrifugation (100 g, 5 min) and cells were resuspended in PBS. Binding of nanoparticles to cells was analysed by cell's side scattering using Novocyte 3000 VYB flow cytometer (ACEA Biosciences, USA).

For confocal microscopy visualization, BSA‐Ge NPs were labeled with Cyanine5 NHS‐ester according to manufacturer protocol. NPs were washed with distilled water from unbound dye by triple centrifugation (15 000 g, 15 min). EMT6/P cells were seeded at glass bottom cell slide and were allowed to grow under a humidified atmosphere with 5% CO_2_ at 37 °C for 24 h. Next, Hoechst 33 342 dye was added to cells at a final concentration of 1 µg mL^−1^. After 1 h of incubation, cell media was removed and BSA‐Ge NPs in phenol red‐free cell medium were added. After 4 h, unbound NPs were removed by gentle rinse with prewarmed cell medium. Confocal microscopy was performed using LSM 980 microscope (Zeiss, Germany) under the following conditions: excitation 405 nm, emission 408–501 nm for Hoechst 33 342 and excitation 639 nm, emission 641–694 nm for Cyanine5.

### Ge NPs Uptake and Degradation in Cells

BSA‐Ge NPs were added to EMT6/P cell suspension (10^6^ cells mL^−1^) to a final Ge concentration of 0.1 and 0.5 mg mL^−1^. The suspensions were incubated for 45 min at 37 °C with gentle mixing every 5 min to prevent cell sedimentation. Then, cells were twice washed from unbound particles by centrifugation (5 min, 100 g) and resuspended with DMEM/F12 medium, containing 10% FBS, at final concentration of 10^5^ cells mL^−1^. The cells were seeded at 12‐well plate by 6×10^4^ in each well. Every 24 h, samples of supernatant were collected. Adherent cells were washed by PBS and treated with Versene solution. Then, samples of supernatants and cells were dissolved by adding nitric acid (10% v/v) followed by ultrasonication, and Ge concentration in solutions was measured by ICP‐MS. The data was normalized by the measured total quantity of the element in the samples.

### In Vitro Cytotoxicity Study

EMT6/P cells at a concentration 10^6^ cells mL^−1^ were incubated with nanoparticles for 45 min at 37 °C in phenol red free DMEM/F12 medium containing 10% FBS. 100 µL of cell suspension was irradiated with 830‐nm laser at 0.7 W power for 1, 5, or 15 min. Then cells were diluted with DMEM/F12 medium containing 10% FBS, seeded into 96‐well plate at a density of 5 × 10^3^ cells per well, and incubated for 48 h at 37 °C under a humidified atmosphere with 5% CO_2_. The cell morphology was examined under a bright‐field DMI 6000B microscope (Leica Microsystems, Germany).

To measure cytotoxicity by MTT test, cell medium was removed from the wells, and 100 µL of 0.5 g L^−1^ MTT solution in serum‐free medium was added to each well. After incubation for 1 h at 37 °C, MTT solution was removed and 100 µL of DMSO was added to each well. Before the measurements plates were thoroughly shaken until formazan crystals were fully dissolved. The absorbance of the samples was measured using a microplate reader at 570 nm, while 670 nm was used as a reference wavelength. The survival percentage was calculated as the signal ratio of the treated cells to the untreated ones.

To measure cytotoxicity by clonogenic assay, after the irradiation cells were seeded into 12‐well plate at a density of 5 × 10^3^ cells per well and incubated for 7 days at 37 °C in 5% CO_2_ with cell medium change every 3 days. When cells formed the colonies, cell medium was removed, and wells were subsequently washed with PBS, 50%, 70% ethanol in PBS, and cells were fixed in 96% ethanol for 15 min. Cell colonies were stained with 1% crystal violet for 30 min, rinsed under tap water to remove excess dye and allowed to dry under the air.

### Photocatalytic Activity Analysis

The photocatalytic activity of Ge NPs was evaluated by degradation of Rhodamine B dye. 200 µg of Ge NPs were incubated with 200 µL of 24 mg L^−1^ Rhodamine B solution in distilled water. Laser irradiation was performed with NIR‐I laser at 1‐W power. After 5, 10, 15, and 30 min solutions were centrifuged at 15 000 g, 10 min and 100 µL of supernatant was collected for analysis. The absorbance spectra were measured using Infinite M1000 microplate reader. As negative controls, Rhodamine B solution was i) incubated under dark; ii) heated at 60 °C; iii) incubated with the same concentration of Ge NPs under dark; iv) irradiated with the same laser at 1‐W power.

### Intracellular ROS Measurement

EMT6/P cells at a concentration 10^6^ cells mL^−1^ were incubated with nanoparticles for 45 min at 37 °C in phenol red free DMEM/F12 medium containing 10% FBS. Then, 100 µL of cell suspension was irradiated with 830‐nm laser at 0.91‐W power for 5 min. Next, cells were treated with 10 µM carboxy‐H2DCFDA for 30 min at 37 °C in darkness. Then, the dye fluorescence was measured using Novocyte 3000 VYB flow cytometer (ACEA Biosciences, USA) with 488‐nm laser excitation and 530/30‐nm band pass filter.

### Pharmacokinetics Study

BSA‐Ge NPs (0.25 mg) in 100 µL PBS were injected into retroorbital sinus of BALB/c mice. Blood samples (10‐20 µL) were collected from the opposite retro‐orbital sinus at 5 s, 5, 15, 30, and 60 min after NP injection. Samples were dissolved in 0.3 mL of 20% nitric acid and diluted with 0.7 mL of water before the ICP‐MS measurement. Blood bioavailability of Ge nanoparticles was quantified as average concentration of the following Ge isotopes: ^70^Ge, ^72^Ge, ^73^Ge, ^74^Ge, and ^76^Ge. Obtained concentrations were normalized to the quantity of blood in the sample, determined by ^56^Fe isotope.

To measure biodistribution of nanoparticles, mice were sacrificed 3 h; 2 days; 1 and 2 weeks, or 1 month after injection, and major organs (liver, spleen, lungs, kidneys, heart, and muscles) were collected and weighed. Then, 50–100 mg of the tissue was dissolved in a threefold volume of 70% nitric acid by 30‐min heating at 80 °C. Then samples were diluted tenfold with distilled water and Ge concentration was measured by ICP‐MS. Data was normalized to the total measured quantity of Ge in collected tissues and presented as %ID g^−1^.

### Photothermal Therapy In Vivo

EMT6/P cells (10^6^) in 100 µL PBS were inoculated subcutaneously into flank region of BALB/c mice. In 6 days, mice with single tumors with an average volume of 150 mm^3^ were chosen for the experiments. The mice were anesthetized, placed on an orbital shaker, and fixed by a tape lent. Mice were divided into three groups: without treatment; with irradiation by 830‐nm laser for 10 min; and with treatment by 1.2 mg of BSA‐Ge NPs and the same laser irradiation parameters. Treatment was performed 20 min after BSA‐Ge NPs injection. The laser beam was focused on the tumor volume and power density was 2 W cm^−2^. Mice were slowly rotated during the irradiation to prevent skin burning. Measurements of tumor volume temperature and whole‐body thermographic images were performed with FLIR C3 camera. The tumor size was measured every day after the treatment by a mechanical caliper and the volume was estimated by the ellipsoid approximation. The survival analysis was performed according to Kaplan‐Meier model by monitoring animals every 1–2 days.

### Photoacoustic Tomography

The TriTom tomograph (PhotoSound Technologies, Inc., Houston, TX, USA) was used to obtain photoacoustic images of Ge NP solutions and mice. For in vitro experiments, the aqueous solutions of BSA‐Ge nanoparticles were placed in the thin capillaries and the capillary ends were sealed with silicon. For in vivo experiments, mice were anesthetized and shaved with a trimmer and depilatory cream before particle injection. Mice were administered with 1.2 mg of BSA‐Ge NPs either intravenously via retro‐orbital injection (in 100 µL PBS) or intratumorally (20 µL). Then, the mouse was placed in a frame holder and paws were clamped with soft fasteners. The head of the mouse was hooked with its front teeth on a horizontal holder, air was supplied directly through the tube. All measurements were carried out using deionized water as the acoustic coupling medium. Both Ge NP solutions and mice were submerged in deionized water‐filled tank alongside the ultrasonic transducer array. The pulsed OPO laser (PhotoSonus, Ekspla, Lithuania) tuned to 830‐nm wavelength was used for photoacoustic excitation. The sample was rotated with 10° s^−1^ velocity during the measurement. The 3D photoacoustic images of both Ge NP solutions and mice were reconstructed with 0.1 × 0.1 × 0.1 mm voxel size using TriTom software (PhotoSound Technologies, Inc., Houston, TX, USA).

### Toxicity Analysis In Vivo

BALB/c mice were injected with 1.2 mg dose of BSA‐Ge NPs into retroorbital sinus. After 3, 15, and 30 days post‐injection 200 µL of mice blood were taken from opposite retroorbital sinus and collected in K3 EDTA tubes (Microvette, Germany). Mice without treatment were used as the negative control. The activity of alanine aminotransferase, aspartate aminotransferase, as well as concentrations of creatinine and blood urea nitrogen were measured by Infinite M1000 PRO spectrophotometer (Tecan, Austria) using commercial kits (Olvex, Russia).

For histopathological analysis, mice were sacrificed at 3 and 30 days post‐injection via cervical dislocation. Major organs (liver, spleen, lungs, kidneys, heart) were collected, incubated for 24 h in 4% PBS‐based formaldehyde solution, dehydrated in a rising ethanol series and embedded in paraffin. Then, tissues were cut into sections of 4‐µm thickness and were stained with hematoxylin and eosin. Slices were photographed using Leica DMI6000B microscope equipped with Leica DMC2900 camera (Leica Microsystems, Germany).

### Statistical Analysis

All experiments were performed at least in triplicates. Data are presented as mean ± standard deviation (SD). Significant differences were determined using one‐way ANOVA with Tukey's post‐hoc test. Significant differences of the survival curves were evaluated using log‐rank test. Statistics were calculated using OriginPro 2021 (Origin Lab) software.

## Conflict of Interest

The authors declare no conflict of interest.

## Supporting information

Supporting Information

## Data Availability

The main data supporting the results of this study are available within the paper and its Supporting Information.
