# Supplementary material for: Laser‐Synthesized Germanium Nanoparticles as Biodegradable Material for Near‐Infrared Photoacoustic Imaging and Cancer Phototherapy
Source: Adv Sci (Weinh). 2024 Mar 22;11(20):2307060. doi: 10.1002/advs.202307060 (PMC11132077; doi:10.1002/advs.202307060)
Supplement: Supplementary file 1 — Supporting Information [file ADVS-11-2307060-s001.pdf]

ADVANCED SCIENCE
Open Access

Supporting Information

for *Adv. Sci.*, DOI 10.1002/advs.202307060

Laser-Synthesized Germanium Nanoparticles as Biodegradable Material for Near-Infrared Photoacoustic Imaging and Cancer Phototherapy

Iaroslav B. Belyaev, Ivan V. Zelepukin, Polina A. Kotelnikova, Gleb V. Tikhonowski, Anton A. Popov, Alina Yu. Kapitannikova, Jugal Barman, Alexey N. Kopylov, Daniil N. Bratashov, Ekaterina S. Prikhozhdenko, Andrei V. Kabashin, Sergey M. Deyev and Andrei V. Zvyagin**

Supporting Information

Laser-Synthesized Germanium Nanoparticles as Biodegradable Material for Near-Infrared Photoacoustic Imaging and Cancer Phototherapy

Iaroslav B. Belyaev, Ivan V. Zelepukin, Polina A. Kotelnikova, Gleb V. Tikhonowski, Anton A. Popov, Alina Yu. Kapitannikova, Jugal Barman, Alexey N. Kopylov, Daniil N. Bratashov, Ekaterina S. Prikhozhdenko, Andrei V. Kabashin, Sergey M. Deyev, Andrei V. Zvyagin**

* E-mails: ivan.zelepukin@ilk.uu.se (I.V.Z.); andrei.zvyagin@mq.edu.au (A.V.Z.)

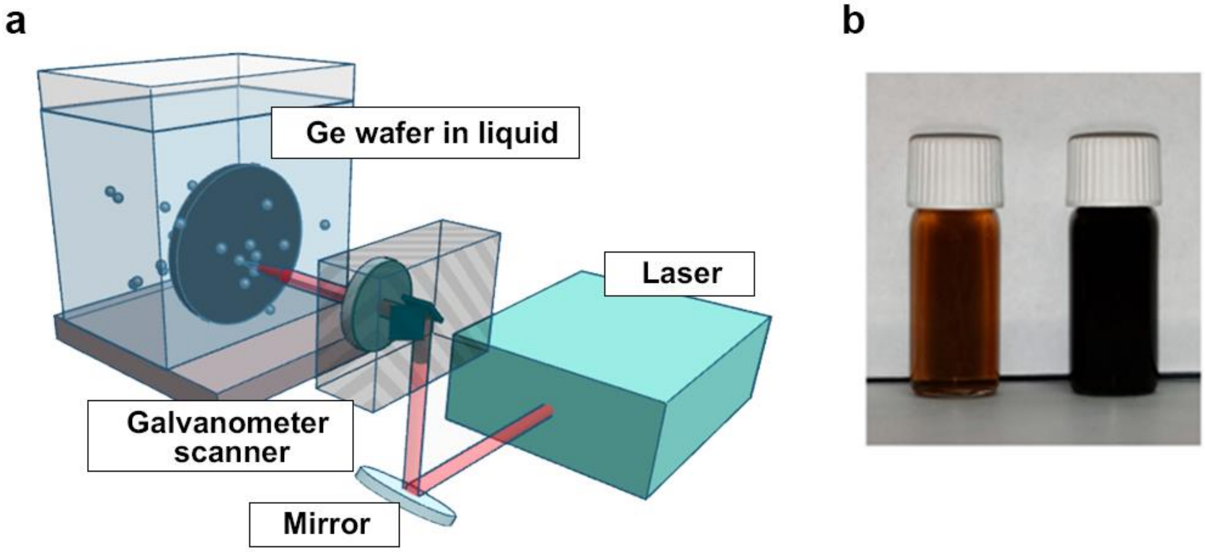

Figure S1. a) Scheme of pulsed laser ablative synthesis of Ge nanoparticles (NPs) in liquids. Beam, generated by femtosecond laser, is directed by a mirror system to a galvanometer scanner equipped with F-theta lens, which irradiates the surface of germanium wafer and hits particles to the surrounding liquid. b) Ge NP colloids in acetone obtained after the process of ablation (left) and 10-fold concentrated solution (right).

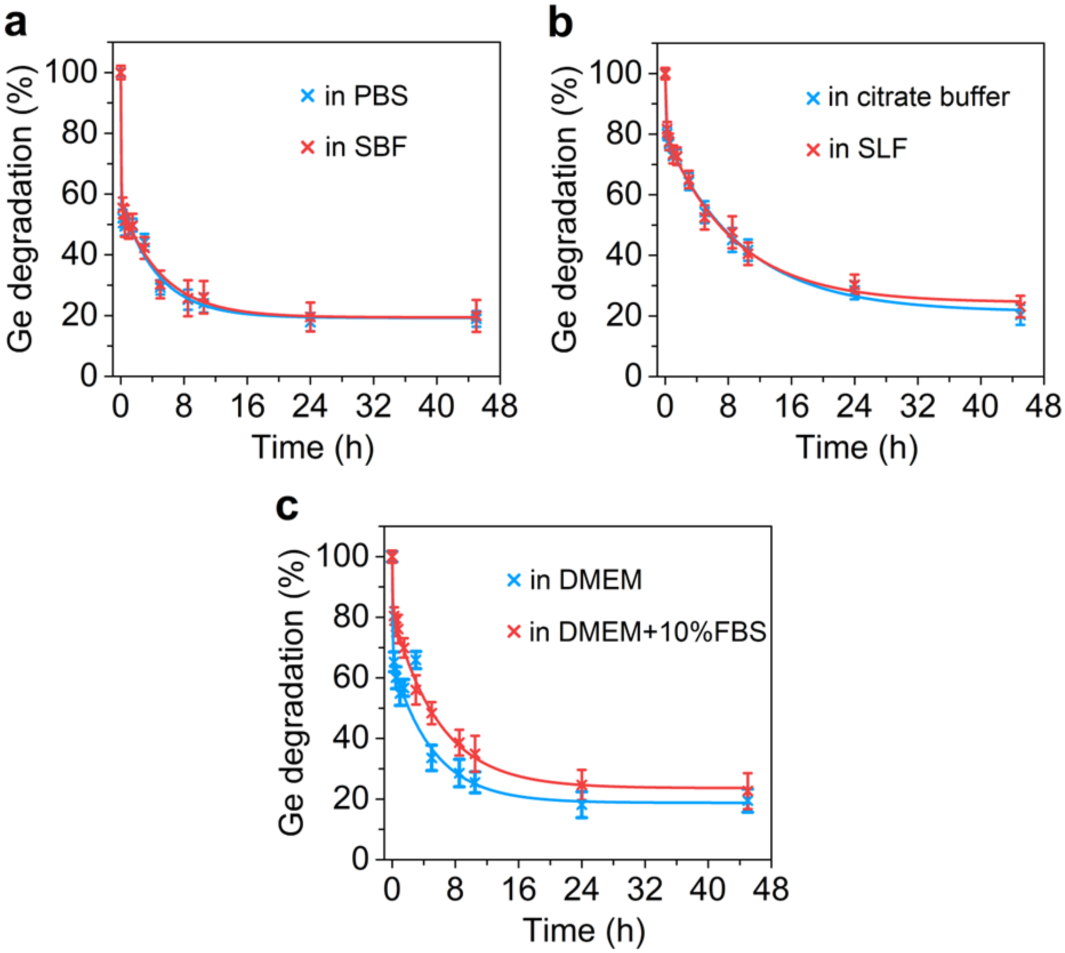

Figure S2. ICP-MS evaluation of Ge NPs degradation in various buffers: a) phosphate buffered saline (PBS, 0.1 M, pH 7.4) and simulated body fluid (SBF, pH 7.4); b) citrate buffer (0.1 M, pH 4.5) and simulated lysosomal fluid (SLF, pH 4.5); c) DMEM/F12 medium and DMEM/F12 supplemented with 10 % fetal bovine serum (FBS). Data are presented as mean ± SD. Solid lines show fitting with biexponential function. $n = 3$ samples.

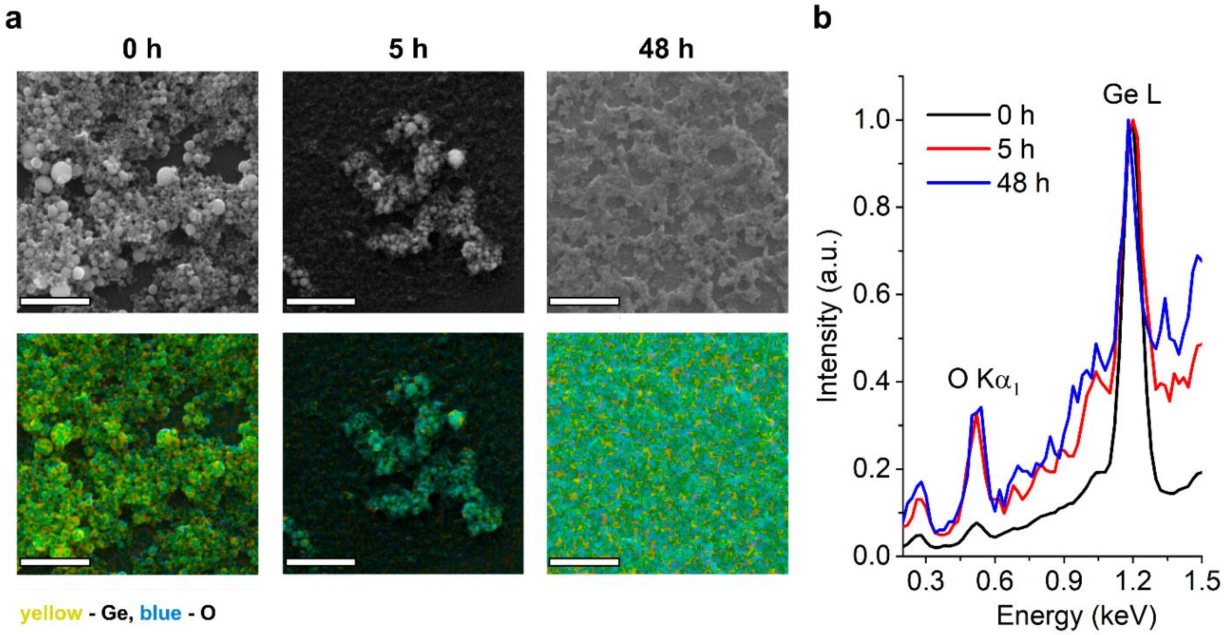

Figure S3. a) SEM images (top) and corresponding energy-dispersive element mapping (down) of Ge NPs incubated in water for different time periods. Scale bars – 1 μm. Elements of Ge and O are marked with yellow and blue colors, respectively. b) Evolution of energy-dispersive spectra of Ge NPs incubated in water for different time periods. The intensities were normalized to the peak signal of Ge L-line.

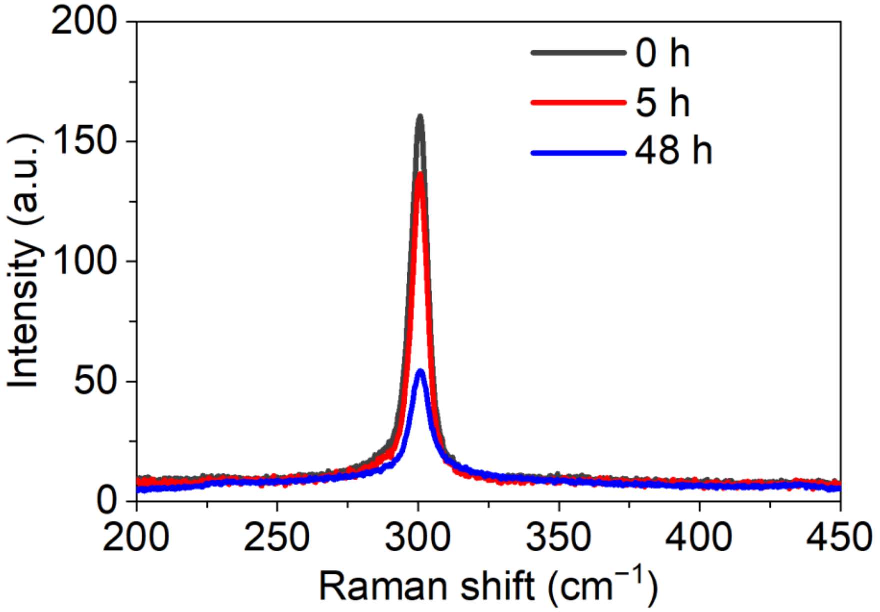

Figure S4. Evolution of Raman spectra of Ge NPs incubated in water for different time periods.

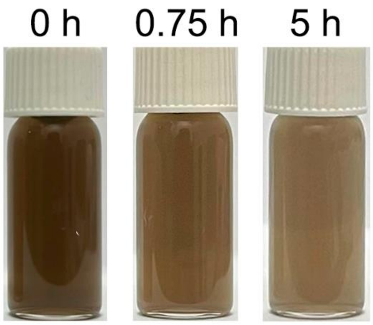

Figure S5. Photographs of Ge NP solutions incubated in water for different periods. Initial concentration of nanoparticles was 100 mg L^{-1}.

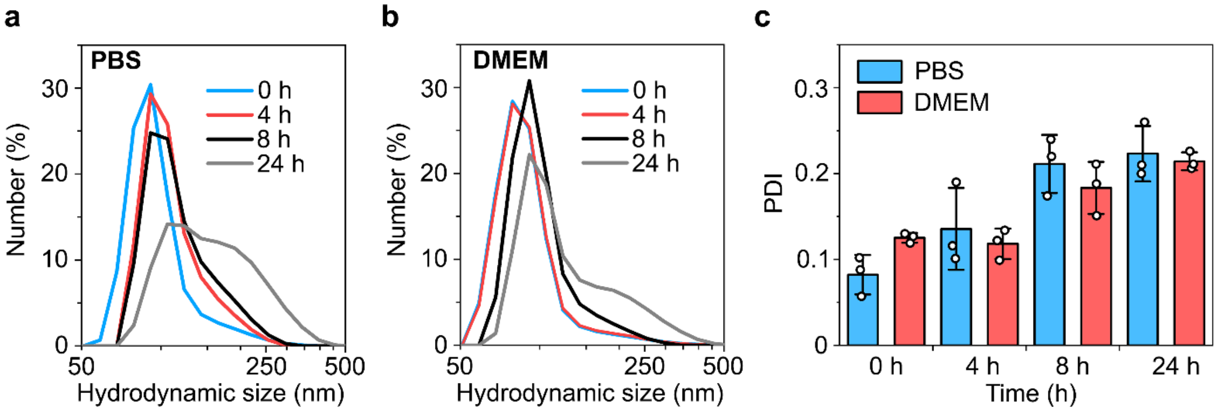

Figure S6. Colloidal stability of BSA-Ge NPs in PBS and DMEM/F12 over time. a,b) Evolution of hydrodynamic size distributions in a) PBS and b) DMEM/F12 medium. c) Change of polydispersity index (PDI) over time. Data in (c) are presented as mean ± SD. $n = 3$ measurements.

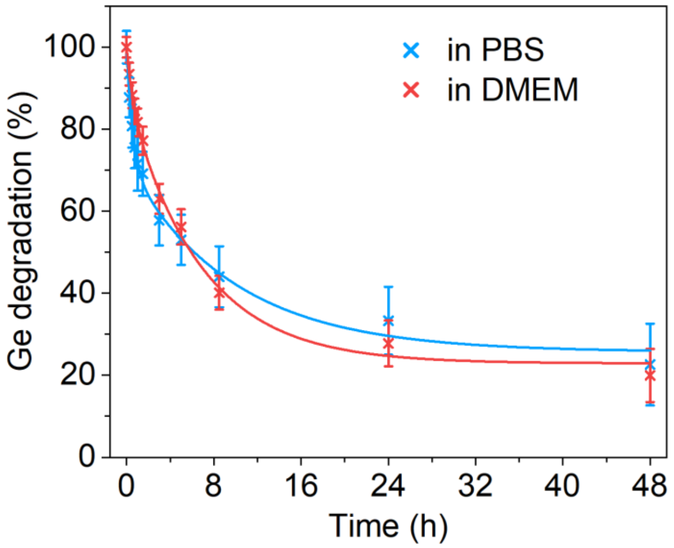

Figure S7. ICP-MS evaluation of BSA-Ge NPs degradation in PBS buffer and DMEM/F12 medium. Data are presented as mean ± SD. Solid lines show fitting with biexponential function. $n = 3$ samples.

Supplementary Note S1. Modeling of light interaction with Ge nanoparticles of variable sizes

The interaction of electromagnetic waves with a homogenous Ge spherical NPs in a water medium was modeled using the Mie solution to Maxwell's equations in Matlab software (2018b version). The extinction, scattering, and absorption cross-sections (σ) for a given wavenumber k of light were defined by the following expressions (equations 1-3):

$$\sigma_{ext} = \frac{2\pi}{k^2}\sum_{j=1}^{\infty}(2j+1)Re(a_j + b_j) \tag{1}$$

$$\sigma_{sca} = \frac{2\pi}{k^2}\sum_{j=1}^{\infty}(2j+1)\left(\left|a_j\right|^2 + |b_j|^2\right) \tag{2}$$

$$\sigma_{abs} = \sigma_{ext} - \sigma_{sca} \tag{3}$$

whith Mie coefficients (equations 4,5):

$$a_j = \frac{m\psi_j(w)\psi_j'(v) - \psi_j(v)\psi_j'(w)}{m\psi_j(w)\xi_j'(v) - \xi_j(v)\psi_j'(w)} \tag{4}$$

$$b_j = \frac{\psi_j(w)\psi_j'(v) - m\psi_j(v)\psi_j'(w)}{\psi_j(w)\xi_j'(v) - m\xi_j(v)\psi_j'(w)} \quad (5)$$

The Ricatti-Bessel functions (equations 6,7):

$$\psi_j(x) = \sqrt{\frac{\pi x}{2}} J_{(j+1/2)}(x) \quad (6)$$

$$\xi_j(x) = \sqrt{\frac{\pi x}{2}} [J_{(j+1/2)}(x) + i\, Y_{(j+1/2)}(x)] \quad (7)$$

The dimensionless parameters for calculation (equations 8-10):

$$m = n_{NPs}/n_{medium} \quad (8)$$

$$v = kr_{NPs} \quad (9)$$

$$w = mv \quad (10)$$

The Bessel functions of the first (J_v) and second (Y_v) order were calculated in the Matlab environment using the built-in functions. The refarctive indices for Ge and water were taken from refractiveindex.info.[1] The calculated cross section for each particle size was normalized to the volume of space occupied by the single nanoparticle with the largest diameter in a simulation (200 nm).

Figure S8 shows modeled data of normilized cross-sections for light extinction, absorption, and scattering in 350–1500 nm wavelength range. Note, that light absorption efficiency in NIR-I and NIR-II regions greatly enhanced for 150–200 nm nanoparticles due to occurence of Mie resonances.

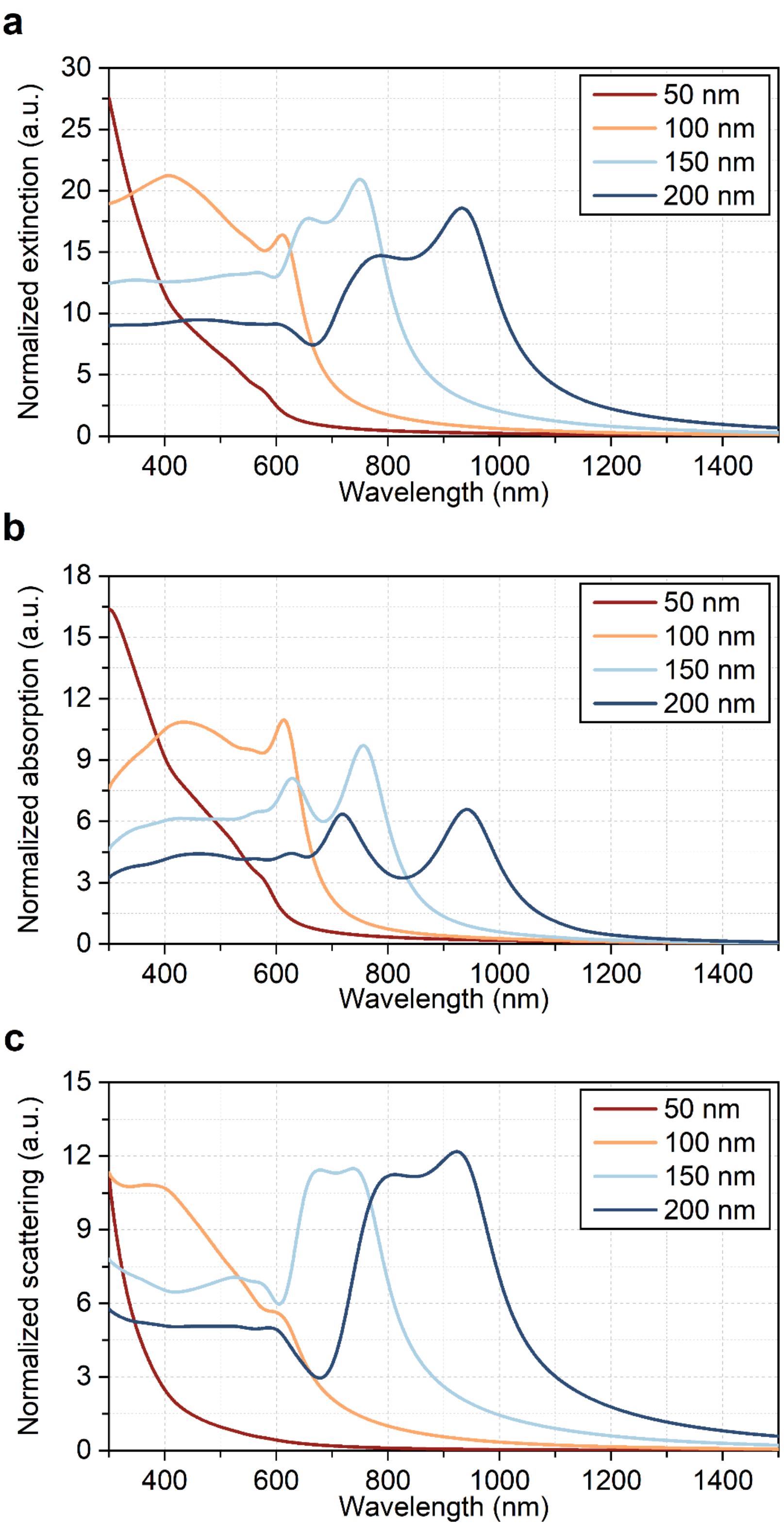

Figure S8. a) Normalized extinction, b) absorption and c) scattering efficiencies of Ge NPs of different sizes.

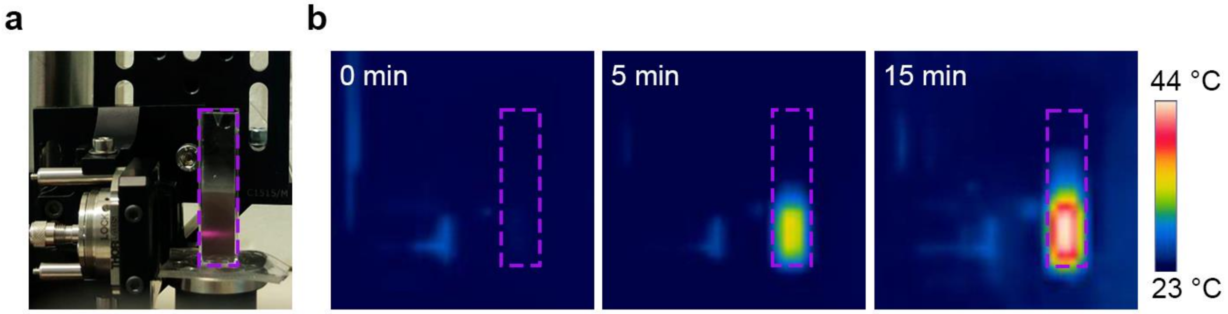

Figure S9. a) Photograph of photothermal heating experimental set-up and b) evolution of thermal map during irradiation in 100 μg mL^{-1} nanoparticle solution. Dashed line contours the cuvette's profile.

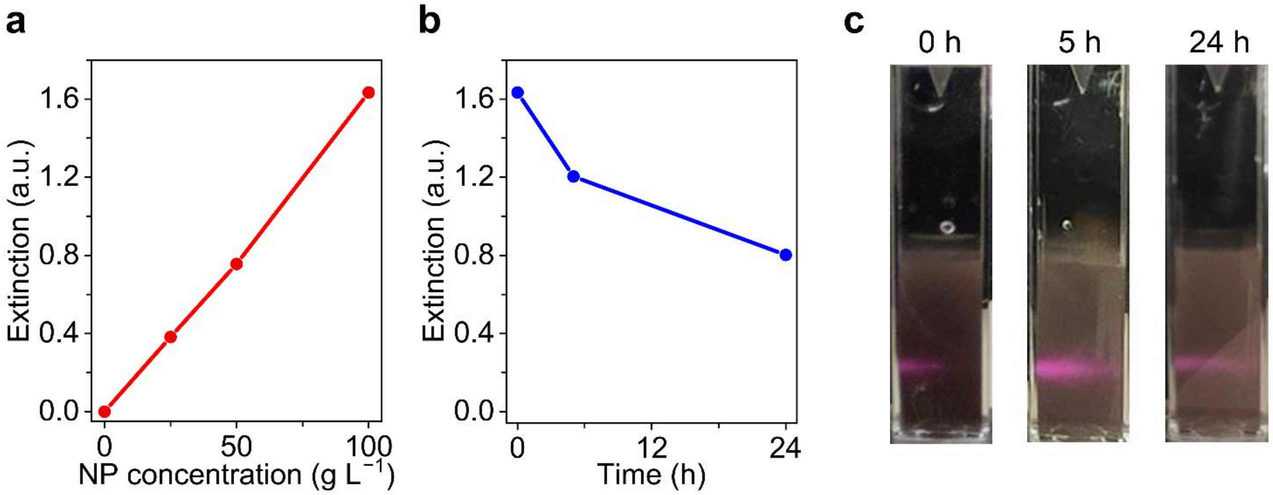

Figure S10. a,b) Change of optical extinction of BSA-Ge NP water solution with concentration (a) and time (b) at wavelength of 830 nm. For time-dependence, 100 μg mL^{-1} is a starting concentration of particles. c) Photographs of same nanoparticle solution during irradiation with 830 nm laser immediately after dispersion in water and after 5 and 24 h.

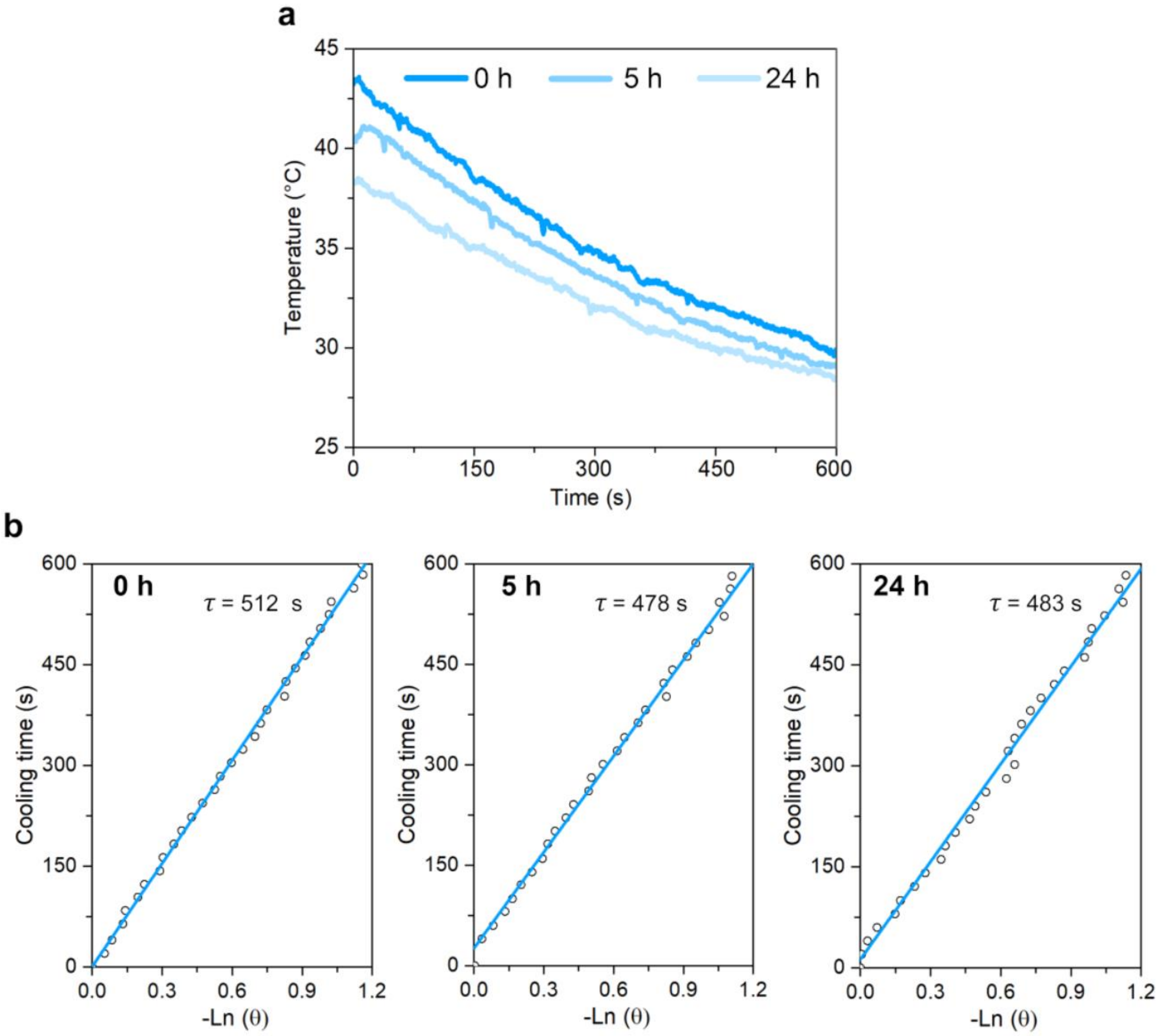

Figure S11. a) Cooling kinetics of BSA-Ge NPs in long-term photothemal stability test. Starting temperatures correspond to the moment of switching off the laser irradiation. b) Corresponding dependencies of logarithm of driving force θ on cooling time. Blue lines show linear fitting with slope τ.

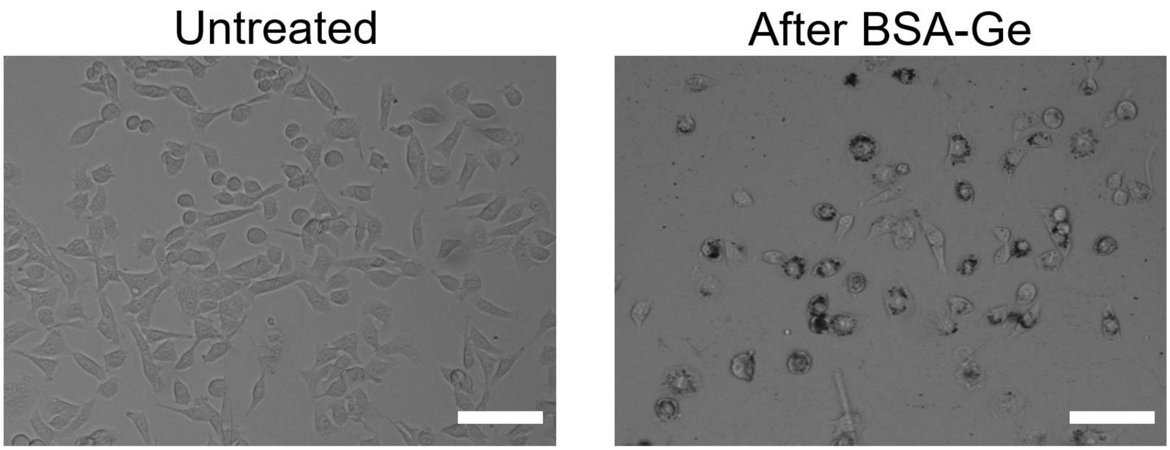

Figure S12. Bright-field microscopy of EMT6/P cell culture before and after treatment with BSA-Ge NPs at concentration 1 g L^{-1}. Scale bar – 100 μm.

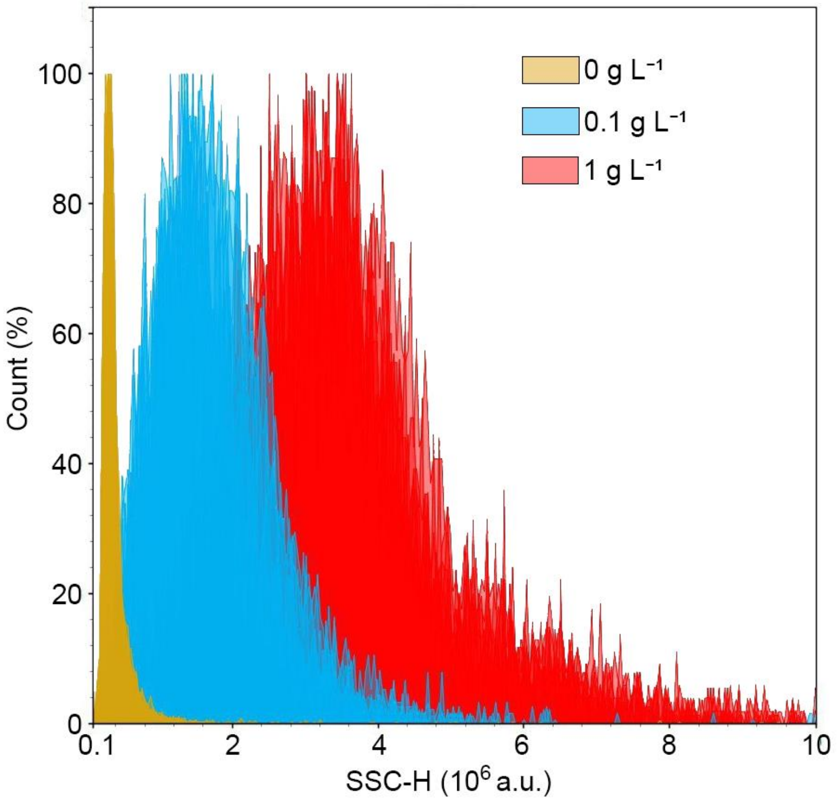

Figure S13. Representative flow cytometry histograms, showing side scattering shift of EMT6/P cells after their incubation with different concentrations of BSA-Ge nanoparticles.

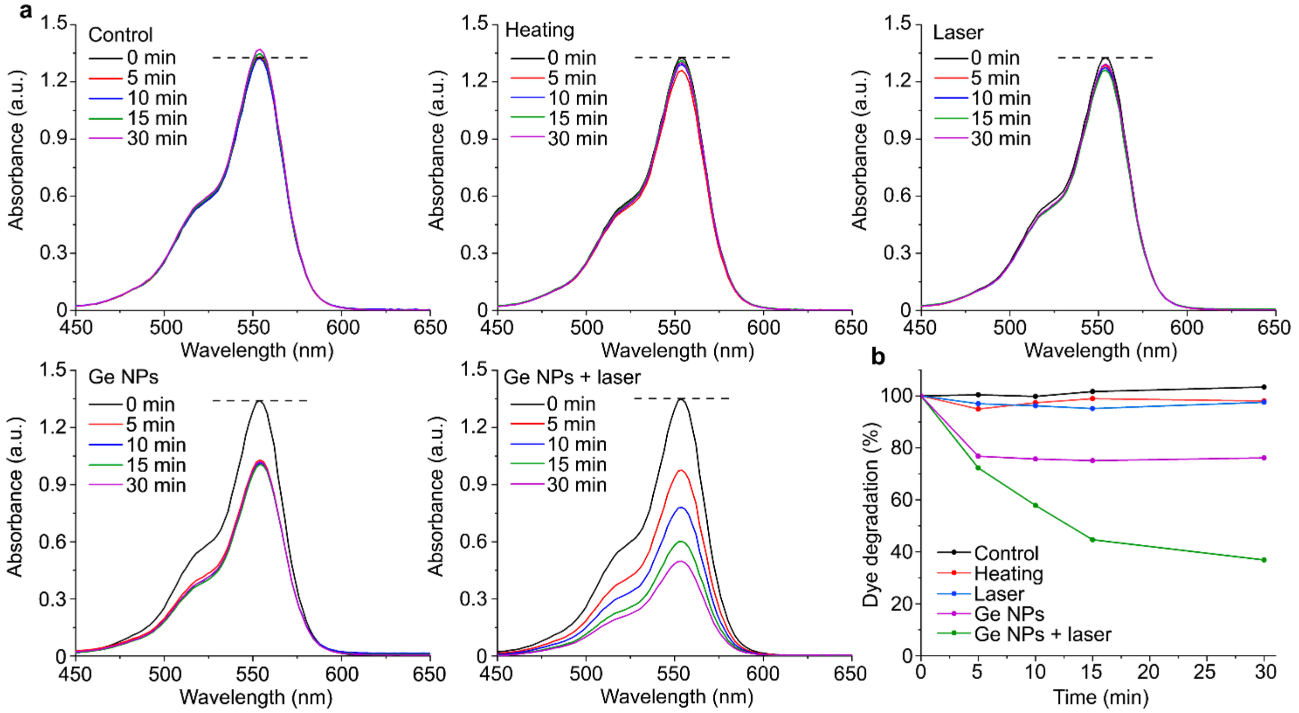

Figure S14. a) Evolution of Rhodamine B absorbance spectra under dark incubation; heating at 60 °C; NIR laser exposure at 1-W power; dark incubation with 1 g L^{-1} Ge NPs; incubation with 1 g L^{-1} Ge NPs under NIR laser exposure at 1-W power. b) Plot of the Rhodamine B dye degradation kinetics.

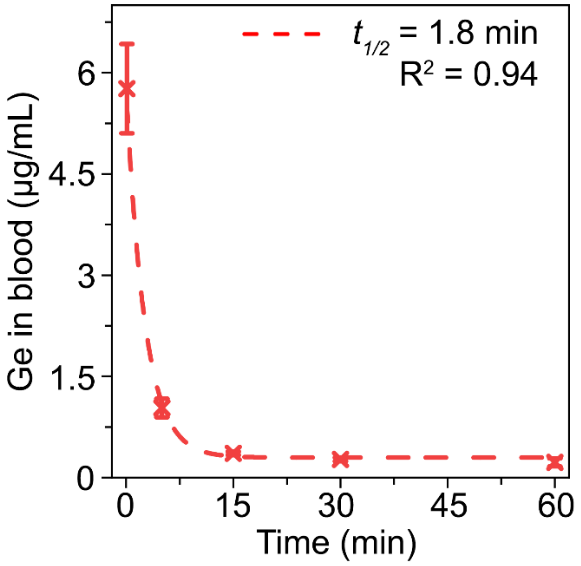

Figure S15. Blood circulation kinetics of BSA-Ge NPs in mice determined by ICP-MS. *n* = 3 animals for each time-point. The red line shows monoexponential fitting.

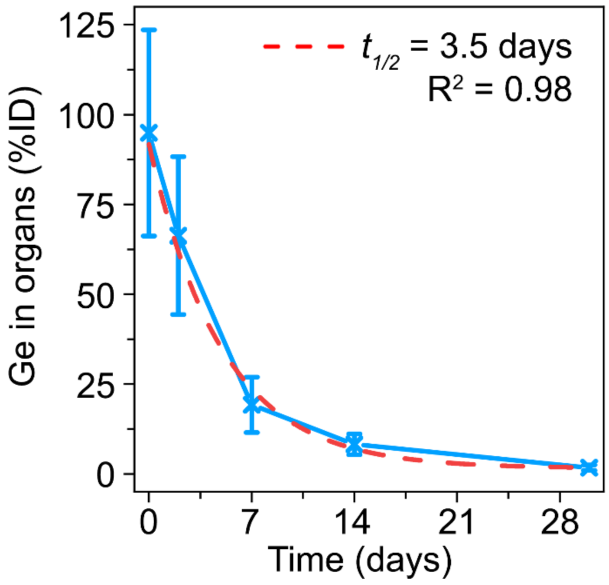

Figure S16. Cumulative elimination kinetics of BSA-Ge NPs from all measured organs (liver, spleen, lungs, kidneys, and heart) of mice. *n* = 3 animals for each time-point. The red line shows monoexponential fitting.

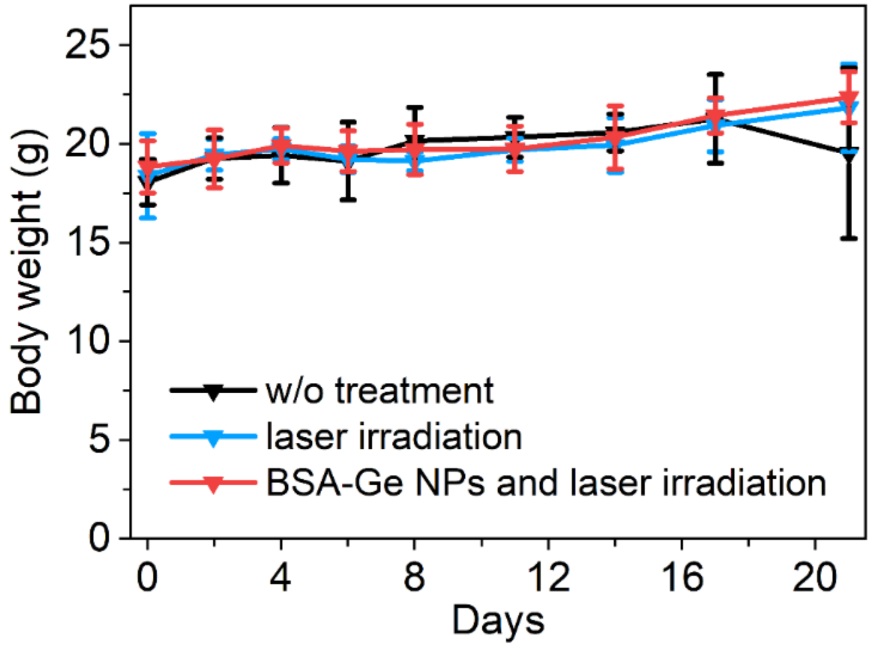

Figure S17. Dynamics of mouse body weight change after photothermal therapy. The number of animals in each group started from *n* = 5 on day 0 and maximally decreased to *n* = 3 with time due to the animals' death. The data are presented as group mean ± standard deviation.

Supplementary Note S2. Comparison of NIR-light extinction and biodegradability of phototheranostic nanomaterials

Notable inorganic nanomaterials, reported for photothermal or photoacoustic biomedical applications, were compared for the values of optical extinction at commonly used NIR-I wavelength of 808 nm based on the published data. We searched for numerical values of extinction coefficient per mass (ε_{808}^{mass}, in units of [L g^{-1} cm^{-1}]) or per mole (ε_{808}^{mol}, [ᴍ$^{-1}$ cm^{-1}]) of nanomaterial. As usually only one of these quantities could be derived, the following formula was applied for conversion:

$$\varepsilon_{808}^{mol} = \varepsilon_{808}^{mass} \times N_A \times \rho \times V_{NP}$$

where N_A is Avogadro's constant, ρ is bulk density, and V_{NP} is expected volume of an average particle. To calculate V_{NP}, morphology and average characteristic dimensions of particles were considered from corresponding references and the following shape approximations were applied: sphere for nanoparticles (NPs), square plate for nanosheets/nanoplates (2D) and QDs, cylinder for nanorods (NRs). For materials with no explicitly reported dimensions, calculation of molar extinction was not applied (NA).

Also, the extinction coefficient per mole of molecular substance (ε_{808}^{mat}) was calculated by relation with molar mass ᴍ [g mol^{-1}]: $\varepsilon_{808}^{mat} = \varepsilon_{808}^{mass} * \text{ᴍ}$.

The analyzed nanomaterials were also classified based on their ability to biodegrade in physiologically-relevant environments, claimed in the reference reporting optical extinction or elsewhere. The following classification from high (A) to low (E) biodegradation potential was used:

A – Degradable in water;

B – Degradable in aqueous solutions at physiological pH;

C – Enzymatically biodegradable;

D – Degradable enzymatically or at physiological pH after the surface coating;

E – Commonly considered non-biodegradable or no proof of biodegradability was found.

The data on the extinction and biodegradability of the selected materials are presented in the Table S1. Materials belonging to categories A, B, C, and D are highlighted in blue.

Table S1. Comparison of extinction coefficients at 808 nm and biodegradation properties of different nanomaterials.

Material	Type	ε_{808}^{mol}, [L mol^{-1} cm^{-1}]	ε_{808}^{mass}, [L g^{-1}cm^{-1}]	ε_{808}^{mat}, [L mol^{-1} cm^{-1}]	Biodegradation class	Ref.
Au	nanorods	5.6×10^9	45.5	8918	E	[2]
Ge	spherical NPs	4.0×10^9	7.9	569	A	This study
Pd	nanosheets	3.5×10^9	68.8	7293	E	[3]
Prussian blue	spherical NPs	1.1×10^9	13.6	11682	B	[2]; [4]
MoS_2	nanosheets	6.2×10^8	28.4	4544	C	[5]; [6]
Nb_2C	nanosheets	5.5×10^8	37.6	7558	D	[7]
Cu_9S_5	nanosheets	4×10^7	11	9516	E	[8]
CuS	spherical NPs	1.7×10^7	8.5	952	D	[9]; [10]
Fe_3O_4	spherical NPs	3.7×10^6	15.1	3488	E	[11]
Mo_2C	quantum dots	2.8×10^6	4.4	898	E	[12]
Pd	spherical NPs	2.6×10^6	2.5	265	E	[13]
Black Phosphorus	quantum dots	2.2×10^5	14.8	458	A	[14]; [15]
WS_2	quantum dots	2.1×10^5	3.3	810	E	[16]
Black Phosphorus	spherical NPs	5.8×10^4	2.1	65	A	[17]; [15]
WS_2	nanosheets	NA	23.8	5879	E	[18]
Mo_2C	nanosheets	NA	18	3654	D	[19]
Ta_4C_3	nanosheets	NA	4.1	3082	E	[20]
FeS	nanosheets	NA	15.5	1349	E	[21]

Supplementary References

[1] a) T. N. Nunley, N. Fernando, N. Samarasingha, J. Moya, C. Nelson, A. Medina, S. Zollner, *J. Vac. Sci. Technol. B* **2016**, *34*, 061205; b) G. M. Hale, M. R. Querry, *Appl. Opt.,* **1973**, *12*, 555.

[2] G. Fu, W. Liu, S. Feng, X. Yue, *Chem. Commun.* **2012**, *48*, 11567.

[3] X. Huang, S. Tang, X. Mu, Y. Dai, G. Chen, Z. Zhou, F. Ruan, Z. Yang, N. Zheng, *Nat. Nanotechnol.* **2011**, *6*, 28.

[4] L. Doveri, G. Dacarro, Y. A. D. Fernandez, M. Razzetti, A. Taglietti, G. Chirico, M. Collini, I. Sorzabal-Bellido, M. Esparza, C. Ortiz-de-Solorzano, X. M. Urteaga, C. Milanese, P. Pallavicin, *Colloids Surf., B* **2023**, *227*, 113373.

[5] T. Liu, C. Wang, X. Gu, H. Gong, L. Cheng, X. Shi, L. Feng, B. Sun, Z. Liu, *Adv. Mater.* **2014**, *26*, 3433.

[6] R. Kurapati, L. Muzi, A. P. R. de Garibay, J. Russier, D. Voiry, I. A. Vacchi, M. Chhowalla, A. Bianco, *Adv. Funct. Mater.* **2017**, *27*, 1605176.

[7] H. Lin, S. Gao, C. Dai, Y. Chen, J. Shi, *J. Am. Chem. Soc.* **2017**, *139*, 16235.

[8] X. Ding, C. H. Liow, M. Zhang, R. Huang, C. Li, H. Shen, M. Liu, Y. Zou, N. Gao, Z. Zhang, Y. Li, Q. Wang, S. Li, J. Jiang, *J. Am. Chem. Soc.* **2014**, *136*, 15684.

[9] G. Ku, M. Zhou, S. Song, Q. Huang, J. Hazle, C. Li, *ACS nano* **2012**, *6*, 7489.

[10] S. Shi, X. Wen, T. Li, X. Wen, Q. Cao, X. Liu, Y. Liu, M. D. Pagel, C. Li, *ACS Appl. Bio Mater.* **2019**, *2*, 3203.

[11] X. Zhang, X. Xu, T. Li, M. Lin, X. Lin, H. Zhang, H. Sun, B. Yang, *ACS Appl. Mater. Interfaces* **2014**, *6,* 14552.

[12] W. Dai, H. Dong, X. Zhang, *Materials* **2018**, *11,* 1776.

[13] B. Rubio-Ruiz, A. M. Pérez-López, T. L. Bray, M. Lee, A. Serrels, M. Prieto, M. Arruebo, N. O. Carragher, V. Sebastián, A. Unciti-Broceta, *ACS Appl. Mater. Interfaces* **2018**, *10*, 3341.

[14] Z. Sun, H. Xie, S. Tang, X.-F. Yu, Z. Guo, J. Shao, H. Zhang, H. Huang, H. Wang, P. K. Chu, *Angew. Chem.* **2015**, *54*, 11526.

[15] Z. Wang, Z. Liu, C. Su, B. Yang, X. Fei, Y. Li, Y. Hou, H. Zhao, Y. Guo, Z. Zhuang, H. Zhong, Z. Guo, *Curr. Med. Chem.* **2019**, *26*, 1788.

[16] Y. Yong, X. Cheng, T. Bao, M. Zu, L. Yan, W. Yin, C. Ge, D. Wang, Z. Gu, Y. Zhao, *ACS nano* **2015**, *9*, 12451.

[17] C. Sun, L. Wen, J. Zeng, Y. Wang, Q. Sun, L. Deng, C. Zhao, Z. Li, *Biomaterials* **2016**, *91*, 81.

[18] L. Cheng, J. Liu, X. Gu, H. Gong, X. Shi, T. Liu, C. Wang, X. Wang, G. Liu, H. Xing et al., *Adv. Mater.* **2014**, *26*, 1886.

[19] W. Feng, R. Wang, Y. Zhou, L. Ding, X. Gao, B. Zhou, P. Hu, Y. Chen, *Adv. Funct. Mater.* **2019**, *29*, 1901942.

[20] H. Lin, Y. Wang, S. Gao, Y. Chen, J. Shi, *Adv. Mater.* **2018**, *30*, 1703284.

[21] K. Yang, G. Yang, L. Chen, L. Cheng, L. Wang, C. Ge, Z. Liu, *Biomaterials* **2015**, *38*, 1.
